# Identification of colorectal cancer progression-associated intestinal microbiome and predictive signature construction

**DOI:** 10.1186/s12967-023-04119-1

**Published:** 2023-06-08

**Authors:** Jungang Liu, Xiaoliang Huang, Chuanbin Chen, Zhen Wang, Zigui Huang, Mingjian Qin, Fuhai He, Binzhe Tang, Chenyan Long, Hong Hu, Shuibo Pan, Junduan Wu, Weizhong Tang

**Affiliations:** 1 Division of Colorectal & Anal Surgery, Department of Gastrointestinal Surgery, Guangxi Medical University Cancer Hospital, Nanning, The People’s Republic of China; 2 School of Public Health, Guangxi Medical University, Nanning, The People’s Republic of China

**Keywords:** Colorectal cancer, 16S rRNA, Intestinal microbiome, Immune infiltration, Staging prediction

## Abstract

**Objective:**

The relationship between intestinal microbiome and colorectal cancer (CRC) progression is unclear. This study aims to identify the intestinal microbiome associated with CRC progression and construct predictive labels to support the accurate assessment and treatment of CRC.

**Method:**

The 192 patients included in the study were divided into stage I-II and stage III-IV CRC patients according to the pathological stages, and preoperative stools were collected from both groups for 16S rDNA sequencing of the intestinal microbiota. Pearson correlation and Spearman correlation coefficient analysis were used to analyze the differential intestinal microbiome and the correlation with tumor microenvironment and to predict the functional pathway. XGBoost model (XGB) and Random Forest model (RF) were used to construct the microbiome-based signature. The total RNA extraction from 17 CRC tumor simples was used for transcriptome sequencing.

**Result:**

The Simpson index of intestinal microbiome in stage III-IV CRC were significantly lower than those in stage I-II CRC. *Proteus*, *Parabacteroides*, *Alistipes* and *Ruminococcus* etc. are significantly enriched genus in feces of CRC patients with stage III-IV. ko00514: Other types of O − glycan biosynthesis pathway is relevant with CRC progression. *Alistipes indistinctus* was positively correlated with mast cells, immune activators IL-6 and IL6R, and GOBP_PROTEIN_FOLDING_IN_ENDOPLASMIC_RETICULUM dominantly. The Random Forest (RF) model and eXtreme Gradient Boosting (XGBoost) model constructed with 42 CRC progression-associated differential bacteria were effective in distinguishing CRC patients between stage I-II and stage III-IV.

**Conclusions:**

The abundance and diversity of intestinal microbiome may increase gradually with the occurrence and progression of CRC. Elevated fetal abundance of *Proteus*, *Parabacteroides*, *Alistipes* and *Ruminococcus* may contribute to CRC progression. Enhanced synthesis of O − glycans may result in CRC progression. *Alistipes indistinctus* may play a facilitated role in mast cell maturation by boosting IL-6 production. *Alistipes indistinctus* may work in the correct folding of endoplasmic reticulum proteins in CRC, reducing ER stress and prompting the survival and deterioration of CRC, which may owe to the enhanced PERK expression and activation of downstream UPR by *Alistipes indistinctus*. The CRC progression-associated differential intestinal microbiome identified in our study can be served as potential microbial markers for CRC staging prediction.

**Supplementary Information:**

The online version contains supplementary material available at 10.1186/s12967-023-04119-1.

## Introduction

Colorectal cancer (CRC) is the third most common malignancy in men and the second most common in women, with the third highest mortality rate among all cancers, and is the leading cause of cancer-related mortality worldwide, and the incidence is increasing significantly in recent years [1, 2]. Studies have shown that patients with early-stage CRC can obtain satisfactory survival through surgery and adjuvant radiotherapy, and their 5-year survival rates are higher compared to those of patients with advanced CRC [3]. At present, early-stage CRC is mainly diagnosed by colonoscopy, which is an invasive test that is not widely available in China, thus reducing the detection rate of early-stage CRC to a certain extent. In addition, due to the heterogeneity of CRC, the lack of specific clinical manifestations and the low sensitivity and specificity of tumor markers, patients are often in progressive or even advanced stages when first diagnosed, and due to factors such as tumor drug resistance and patient compliance, the best time for treatment is missed, resulting in poor prognosis. Therefore, it is extremely important to find a non-invasive screening method for early detection of CRC.

The mechanisms of CRC are still undergoing, and it is generally accepted that the development of CRC is related to epigenetic, dietary, environmental and metabolic factors. The human intestinal microbiota consists of more than 1000 microorganisms, including microbiome, fungal and viral groups, with more than 100 times the number of genes than humans [4]. The stability of the number and species of normal human intestinal microbiome plays a crucial role in shaping the intestinal epithelium, maintaining immunity and defending against pathogenic bacteria. Evidence is accumulating that intestinal microbiota is involved in the development of human malignancy as a key factor. Metabolites of the intestinal microbiota can induce oncogenic stress responses, such as the transition growth of *Porphyromonas* secretes butyrate analogs that increase the expression of senescence-inducing genes (p16INK4a and p21Cip1/Waf1) and SASP factor genes (IL-1 and IL-6), accelerating cellular senescence, leading to increased DNA damage and p53 phosphorylation formation, which induces the development of CRC [5]. It is shown that the microbiome plays a pivotal role in the development of many human diseases through its involvement in human metabolic and immune processes. Previous research evidence suggests that the human intestinal microbiome is one of the important factors associated with CRC. A variety of microorganisms can trigger intestinal inflammatory responses and oncogenic signals to promote CRC development, and when the intestinal microbiota is displaced or functionally abnormal, it can disrupt intestinal epithelial cells and induce CRC development by participating in inflammatory responses, host organism immunity, and production of oncogenic substances [6]. Besides, it is also demonstrated that enriched gut microbiome differs between older CRC patients and younger CRC patients at different clinical stages and tumor locations, the predominance of DNA binding and RNA-dependent DNA biosynthesis processes in younger CRC patients has been demonstrated when studying metabolic alterations in the intestinal microbiome system [7]. Those evidence indicated a significant interaction between the intestinal microbiome and the clinicopathological features of CRC can enhance the CRC cells’ invasive and proliferation capacity via activating the transcriptional and translational biosynthetic processes. What’s more, it has been shown that APC mutations in the host are closely associated with changes in intestinal microbiome and serum metabolites for predicting CRC development in adenomatous polyps of the intestine [8]. Liu et al. have showed that a significant decrease in traditional beneficial bacteria such as *Lactobacillus* and *Bifidobacterium* and a significant increase in oncogenic bacteria such as *Enterobacteriaceae* and *Fusobacterium nucleatum* in CRC patients, which tested that endotoxin (LPS), D- In addition, elevated levels of endotoxin (LPS), D-lactate and diamine oxidase (DAO) were confirmed to be associated with dysregulated fecal microbiota and intestinal mucosal barrier dysfunction in early CRC patients [9]. This kind of changes in the number and type of intestinal flora may work together to involve in CRC process and provide a new non-invasive test and precise treatment for a specific flora in CRC patients. Hence, in order to clarify which of intestinal flora that are decreasing or increasing in CRC patients to better understand the mechanism of CRC, the fecal of CRC patients was to collected to further analyses. Interestingly, there are few relevant studies on the changes of intestinal microbiome in the stool of patients with early and advanced CRC.

In the present study, in order to search for intestinal microbiome associated with the development of CRC to find new biomarkers for the diagnosis of early CRC and to provide new strategies for the prevention and treatment of early CRC, a total of 192 samples with CRC patients’ fecal are submitted to16S rDNA gene sequencing and are analyzed the differences between early and advanced CRC patients, which may shed light on the pathogenesis of CRC in the intestinal microbiome.

## Methods

### Sample collection

198 CRC patients’ fecal samples in Guangxi Medical University Cancer Hospital were qualified during selected 236 fecal samples during 2021.01.01 to 2021.12.31. Among them, there were 192 samples with certain TNM stage for our analysis. Inclusion criteria: (1) Surgical treatment with clear pathological stage according to ACJJ CRC staging guidelines; (2) No combination or no previous other malignancies; (3) Exclusion of other intestinal diseases and no acute comorbidities such as complete intestinal obstruction, intestinal perforation, or pelvic abscess; (4) Patients did not receive any Anti-tumor treatment, such as surgery, chemotherapy, radiotherapy, immunotherapy and Chinese herbal medicine, etc.; (5) No antibiotics and intestinal microecological agents were used in the past 1 month; (6) No impairment of consciousness or other cognitive dysfunction. Based on the postoperative pathological staging of CRC patients included in the study, stages I-II were defined as early colorectal cancer, while stages III-IV were defined as advanced colorectal cancer. Before treatment, the patients' fecal samples were taken in sterile ice boxes, dispensed in 2 mL EP tubes and stored in a refrigerator at -80 ℃. The study was approved by the medical ethics committee of our hospital, and all study subjects signed an informed consent form.

### 16S rDNA gene-sequencing and microbiome analysis

Take 200 mg of fecal sample and add 2 mL of EX buffer. Take 1.2 mL of fecal lysate, heat at 70 ℃ for 5 min, centrifuge at 1,500 r/min for 1 min, take 200 μL of supernatant in a microcentrifuge tube containing 1.5 mL of proteinase K; incubate at 70 ℃ for 10 min, add 200 μL of 75% ethanol and mix well; pass 600 μL of lysate through a QIAamp centrifuge column and centrifuge; add 500 μL of LBuffer AW2 to the column to elute DNA. The DNA was eluted using primers (341F:5′-CCTACGGGNGGCWGCAG-3′;805R:5′-GACTACHVGGGTATCTAATCC-3′) targeted to the V3 and V4 high variant regions of the 16S rDNA gene on an ABI 2720 Thermal Cycler for 16S rDNA gene amplification. Normalized equimolar concentrations of each amplicon were then pooled and sequenced on the Miseq PE250 (Illumina) platform using 2 × 250 bp chemistry according to the manufacturer's instructions. DNA concentrations of sequenced libraries were measured by Qubit 3.0 fluorometer DNA HS analysis (Thermo Fisher Scientific) and specifically differentiated in Invitrogen Qubit3.0 lines. The number and size of sequencing libraries were analyzed using an Agilent BioAnalyzer 2100 (Agilent), and comparative analysis of colony richness was performed in patients with stage I-II and III-IV CRC. Subsequent raw data analysis of 16S rDNA sequencing was performed with Quantitative In-sights Into Microbial Ecology version 2 (QIIME2).

LEfSe and QIIME2 were used for analysis by R software with R package (heatmap, ggplot2).The LEfSe analysis was used to screen species for inter-group differences through Lefse Software v1.0.0, firstly by using the non-parametric Kruskal–Wallis rank sum test in multiple sample groups to screen for species with significant differences in abundance between subgroups; then, based on the significantly different species obtained, the Wilcoxon rank sum test was used in pairs to perform an inter-subgroup difference analysis, and finally, linear discriminant analysis (LDA) was used to assess the effect value of each significantly different species. Intra-group microbiome diversity was analyzed by α diversity, and we used Chao1 and ACE indices to describe the abundance of intestinal microbiome and Shannon and Simpson indices to describe the abundance and homogeneity of intestinal microbiome. And for inter-group colony diversity was analyzed by β diversity. The variability in species composition of the gut flora of each sample in the same ecology was measured by β diversity, and ADONIS analysis in beta diversity analysis was performed by vegan package v2.5.6. The Partial Least squares-discriminant Analysis (PLS-DA) of fecal intestinal flora was performed by the package mixOmics v6.6.2. Developed in 2013 by Langille et al., PICRUSt (Phylogenetic Investigation of Communities by Reconstruction of Unobserved States) is a bioinformatics software which can be used to predicting the function of metagenomes based on 16S sequencing data, especially for the functional prediction of human microbial community with high accuracy [10]. PICRUSt2, an updated version of PICRUSt, was implemented to predict the enriched KEGG pathways between microbial community in patients with stage I-II and stage III-IV CRC in this study.

### XGBoost model (XGB) and Random Forest (RF) models construction

The XGBoost (eXtreme Gradient Boosting, XGB) model and the Random Forest (RF) model, two kinds of extensive machine learning methods, were applied to identify the intestinal microbiome to predict stage I-II and stage III-IV in CRC patients, respectively. XGB can improve the efficiency and scalability of the model by avoiding model over-fitting, reducing the complexity of the model, while RF is evaluated synthetically by combining multiple decision trees, and the generalization error converges continuously as the number of trees increases [11–13]. Python Scikit learning (https://scikit-learn.org/stable/; v, 0.18) was applied to the toolkit used to construct and evaluate the models, and default parameters were used for modeling. The subject operating curve (ROC) and area under the curve (AUC) were used to assess the accuracy of the constructed models by applying cross-validation tests to assess the stability of the models.

### Transcriptome sequencing

Total RNA was isolated from 17 CRC tumor samples by the Trizol® Total RNA Extraction Kit according to the instructions. Then, we checked the purity of the RNA by micro UV spectrophotometer when the integrity of RNA was tested. Besides, cDNA libraries were constructed according to the instructions of the RNA-seq Sample Preparation Kit (VAHTS™ Stranded mRNA-seq Library Prep Kit for Illumina®) after removing the rRNA. In order to assessed the quality of sequenced raw data which was performed by Illumina NovaSeq 6000, FastQC was applied for this purpose. All the samples valid data were firstly matched to the reference genome (version: hg38) using HISAT2 for valid data. The expression of genes was evaluated. The TPM (Transcripts Per Million) calculated for each gene was used as the expression abundance of that gene after the expression of genes was assessed using StringTie and known gene models, and the TPM (Transcripts Per Million) calculated for each gene was used as the expression abundance of that gene.

### Differential bacterial flora and the correlation with tumor microenvironment

CIBERSORT, the most cited tool for the analysis of immune cell infiltration estimation (http://cibersort.stanford.edu), is used for the analysis of tumor infiltration of immune cells, immune targets, etc. The CIBERSORT algorithm, using microarray data to construct a feature matrix, can transform the TPM matrix into a relative content matrix of 22 immune cells, including immune cells with different cell types and functional states such as B cells, CD4 + T cells, CD8 + T cells, neutrophils, macrophages, dendritic cells, NK cells, immune targets and chemokines. We transformed the transcriptome sequencing data into the relative content matrix of 22 immune cells using the CIBERSORT analysis tool and R software with ‘CIBERSORT R script v1.03’.

### Identification of differential pathway

The analysis of variance of The KEGG pathways and GO items were all performed using the Single Sample Gene Set Enrichment Analysis (ssGSEA) algorithm, which a rank-based method that calculates a score for the absolute enrichment of a specific gene set for each sample. After downloading the gene set file in gmt format (c2.cp.kegg. v2022.1. Hs. symbols. gmt, c5. go. v2022.1. Hs. symbols. gmt) from the official website of GSEA (https://www.gsea-msigdb.org/gsea/index.jsp), we used the “ssgsea” algorithm to calculate the gene set score matrix for each sample with the GSVA package v1.46.0. Then, the functional pathways were analyzed by the limma algorithm with the TCGAbiolinks package v2.25.3. GO analysis includes three levels: biological process (BP), molecular function (MF), and cellular component (CC). The significance threshold for differentially expressed genes is: p value < 0.05 and | log2FC |> 0.

### Statistical analysis

T-Test and Pearson Chi-square for the data of clinical baseline table were performed by SPSS software v23.0. Pearson correlation analysis was performed between intestinal flora, immune cell abundance, immune-related genes and among dominant flora in both CRC groups by Hmisc package v4.7-1. Spearman correlation analysis was performed between intestinal flora and BP items and MF items by ggcorrplot package v0.1.4, between intestinal flora and KEGG pathways by Hmisc package v4.7-1. R software (version 4.20) was applied for statistical analyses package ggplot2 v3.4.0, pheatmap v1.0.8, Igraph package v1.3.5 and Cystoscope software Version 3.7.2 were used for graph visualization [14, 15]. Differences were considered statistically significant at P < 0.05.

## Result

### Basic information and clinical characteristics of recruited colorectal cancer patients

After inclusion and exclusion, this study had completed the collection of stool samples from 236 patients with colorectal cancer (CRC)before treatment, and finally 198 qualified fecal samples were received for 16S rRNA sequencing. Of these, 192 CRC patients had certain information of TNM stage, including 62 CRC patients with stage I-II and 130 CRC patients with stage III-IV. As illustrated in the Table 1, there was no statistically significant difference in age between CRC patients with stage III-IV and those with stage I-II, the proportion of male CRC patients was significantly higher than that of female CRC patients (Pearson Chi-square, P = 0.003). The proportion of CRC  patients with stage III-IV accompanied with vascular invasion (Pearson Chi-square, P = 0.033) and perineural invasion (Pearson Chi-square, P = 0.003) were significantly higher than those of CRC patients with stage I-II, suggesting that stage III-IV CRC is more malignant than stage I-II.

**Table 1:** Demographic and clinical characteristics of CRC patients stratified by CRC progression-related condition

		CRC patients with advanced stage (TMN 3–4) (n = 130)	CRC patients with early stage (TMN 0–2) (n = 62)	P value	Test
Age (years, mean (SD))		57.52 ± 11.96	59.60 ± 10.16	0.240	T-Test
Age (%)	≥ 60	57 (43.80)	27 (43.50)	0.969	Pearson Chi-square
	< 60	73 (56.20)	35 (56.50)		
Gender (%)	male	67 (51.50)	46 (74.20)	0.003**	Pearson Chi-square
	female	63 (48.50)	16 (25.80)		
LVI invasion (%)	YES	32 (43.20)	15 (25.40)	0.033*	Pearson Chi-square
	NO	42 (56.80)	44 (74.60)		
perineural invasion (%)	YES	49 (67.10)	24 (41.40)	0.003**	Pearson Chi-square
	NO	24 (32.90)	34 (58.60)		

The "*" in the upper right corner of the P value indicates the size of the P value: none* for P value ≥ 0.05. * for 0.01 ≤ P < 0.05. ** for 0.001 ≤ P < 0.01

### Comparison of intestinal microbiome between colorectal cancer patients at stage I-II and stage III-IV

**Fig. 1:**
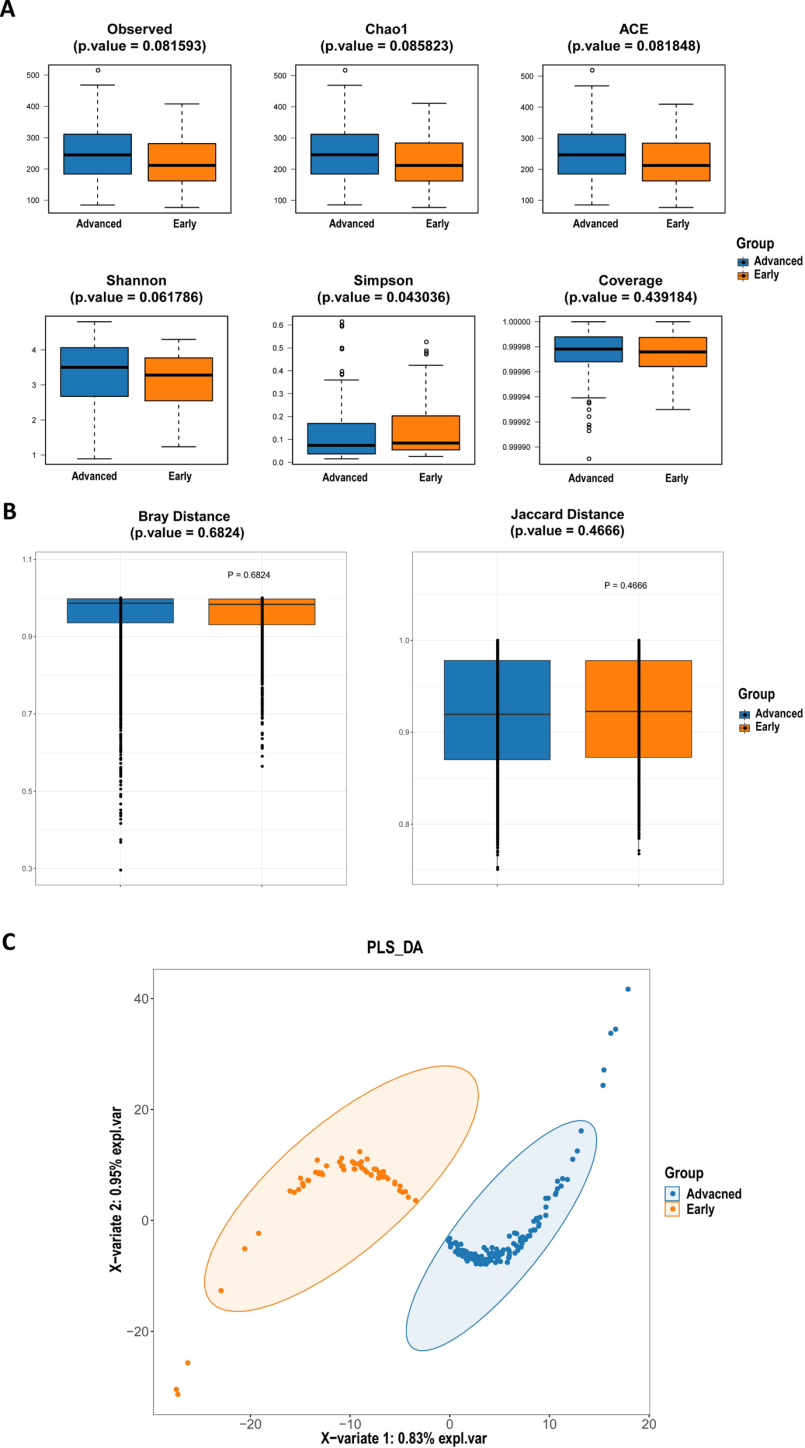
Comparison of diversity index of intestinal microbiome in CRC patients with stage I-II and III-IV. **A** Comparison of α diversity index of intestinal flora in CRC patients with stage I-II and III-IV. **B** Comparison of β diversity index of intestinal flora in CRC patients with stage I-II and III-IV. The horizontal coordinates indicate the group, the vertical coordinates indicate the value of community diversity index of the samples in this group, and the color indicates the group. **C** PLS-DA analysis of intestinal flora in the stage I-II group versus the stage III-IV group of CRC patients. The dots represent each intestinal flora sample, the color indicates the group, the scale of horizontal and vertical axes indicates the relative distance of each sample, and X-variate 2 and X-variate 1 represent the factors affecting the shift of intestinal flora composition in stage I-II group and stage III-IV group of CRC patients, respectively

Firstly, we compared the intestinal microbiome diversity between CRC patients with stage I-II and stage III-IV group based on α diversity and β diversity index. Fig. 1A showed six α diversity indices of intestinal microbiota samples of CRC patients with stage I-II in comparison to that of CRC patients with stage III-IV, only the Simpson index was significantly higher (Wilcoxon, P = 0.043). The β diversity of CRC patient with stage I-II and stage III-IV group was shown in Fig. 1B. There were no significant differences statistically in Bray and Jaccard distance index between the two groups. (Additional file 7: Tables S1, Additional file 8: Tables S2). Partial Least Squares Discriminant Analysis (PLS-DA) analysis suggested that CRC patients with stage I-II and stage III-IV could be distinguished into distinct clusters (Fig. 1C). These results indicated that the richness and diversity of fecal microbial communities in stage III-IV CRC was significantly higher than that of stage I-II CRC, and there was a clear between-group difference in the composition of intestinal microbiome between them.

### Identification of intestinal microbiome associated with CRC progression

**Fig. 2:**
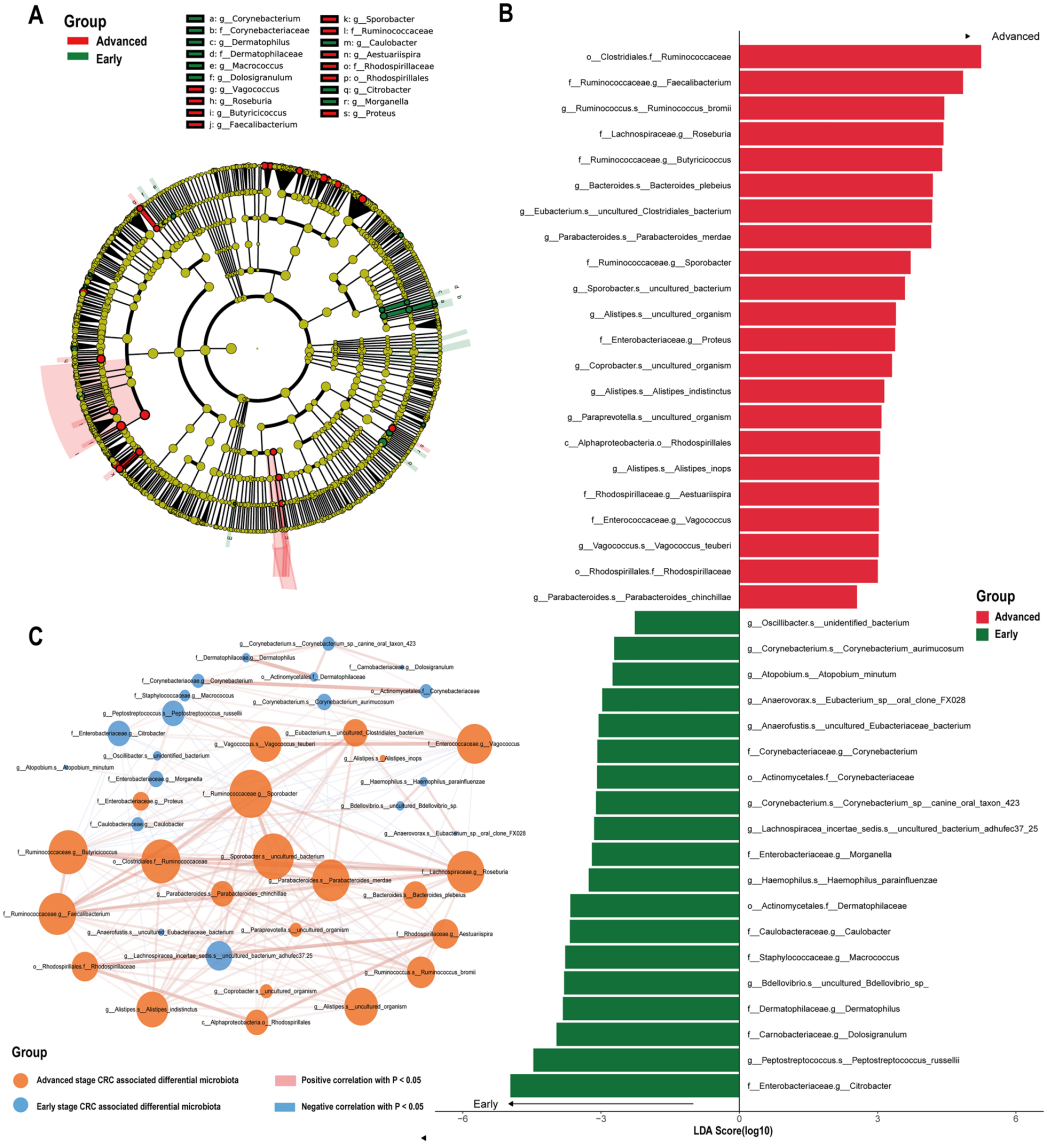
Differential analysis of intestinal flora of CRC patients in stage I-II group and stage III-IV group. **A** cladogram by LEfSe analysis. Node size represents species abundance size, which is proportional to species abundance size. Node color represents grouping, yellow nodes in the tree branch indicate species with no significant abundance differences between groups; red nodes indicate species with significantly higher abundance in stage III-IV groups, and green nodes indicate species with significantly higher abundance in stage I-II groups. Each layer of nodes indicates phylum/class/order/family/genus/species from inside-out, and the annotation of each layer of species markers indicates phylum/class/order/family/genus/species from outside-in. **B** LDA bar graph based on 16S rRNA gene sequencing. The color of the bar represents the group, the horizontal coordinate is the LDA score (after log10 treatment), the vertical coordinate indicates the species with significantly higher abundance in the group, and the length of the bar represents the size of the LDA score value. **C** Network diagram of the correlation of differential intestinal flora associated with phase I-II groups and phase III-IV groups. Each node represents each species, node color represents grouping, node size represents the number of edges connected to the node, the larger the node, the more the number of edges connected to the node, the connecting line represents the existence of significant correlation between two nodes, Spearman correlation coefficient value below 0 (negative correlation) indicates blue line, Spearman correlation coefficient value greater than 0 (positive correlation) indicates red line The thicker the line, the greater the Spearman correlation coefficient between the two nodes

In order to seek out potential biomarkers of intestinal microbiome associated with CRC progression, we performed LDA Effect Size (LEfSe) analysis on fecal microbiota of CRC patients with stage I-II and III-IV respectively, and filtered intestinal microbiota with significant difference in richness between stage I-II and stage III-IV CRC. Through the analysis, we found a total of 41 bacteria with significant differences in abundance statistically. Among them, the abundance of a total of 19 bacteria in stage I-II CRC group was significantly higher than that in stage III-IV group, and the abundance of 22 bacteria of stage III-IV CRC patients was significantly higher than that of stage III-IV CRC patients (Wilcoxon, P < 0.05, as shown in Additional file 9: Table S3 and Fig. 2A, B). The Linear Discriminant Analysis (LDA) bar plot of Fig. 2B exhibited the top ten bacteria with the highest LDA scores in each group, where higher scores indicated greater influence of the species (LDA scores were processed by log10).

In addition, in order to explore the interaction between the different intestinal microbiome between the stage I-II and stage III-IV CRC patients, we depicted the correlation graph between the dominant microbiota of the stage I-II groupa and stage III-IV group (Fig. 2C). Herein, *f__Ruminococcaceae.g__Sporobacter*, *g__Sporobacter.s__uncultured_bacterium*, *f__Ruminococcaceae.g__Butyricicoccus*, *o__Clostridiales.f__Ruminococcaceae and g__Parabacteroides.s__Parabacteroides_merdae* were most connected to other nodes, which indicated that these five intestinal microbiome were most closely related to other dominant bacteria. Moreover, there was a significant negative correlation between the Significantly enriched microbiota in stage III-IV CRC and most of the dominant microbiota enriched in stage I-II CRC. These results manifested that there might be a competitive relationship among these dominant microbiota enriched in both groups.

### Function prediction of intestinal microbiome in CRC patients with stage I-II group and stage III-IV

**Fig. 3:**
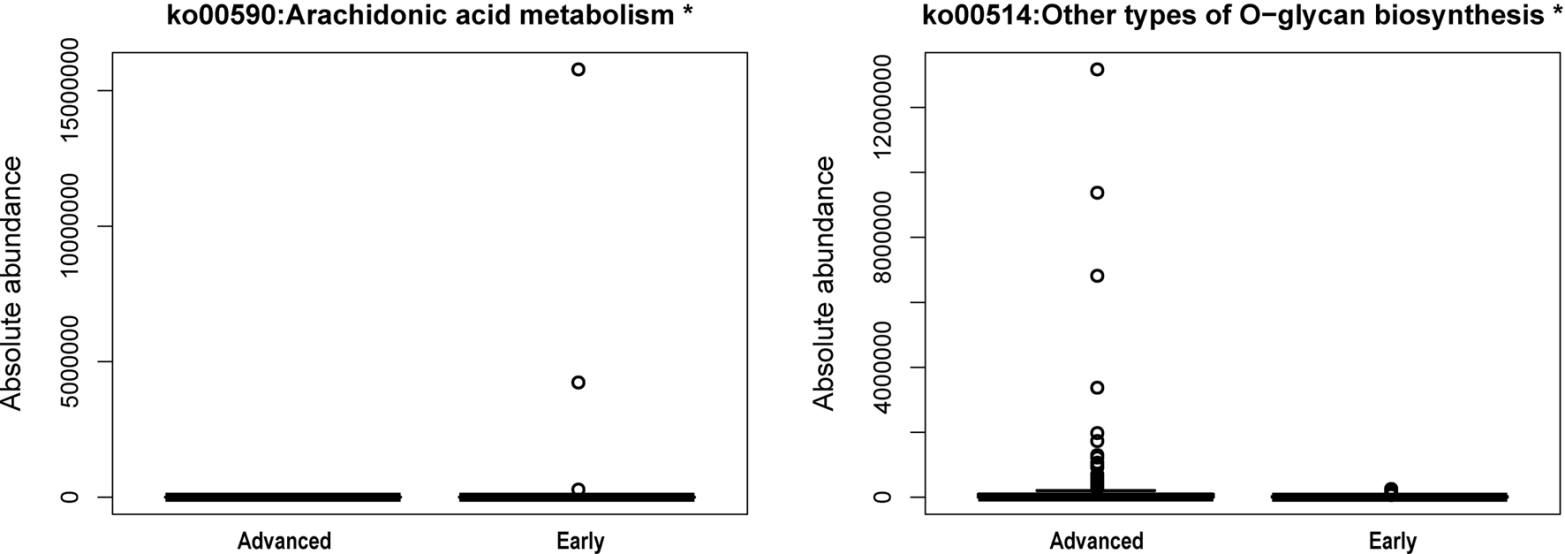
Box plots of KEGG functional abundance in CRC patients in stage I-II group and stage III-IV group. Horizontal coordinates indicate groups, vertical coordinates indicate predicted abundance values for that pathway in each sample, boxes indicate the 25th–75th percentiles, and the center marker indicates the median; black bars are 1.5 times the interquartile range

To explore the biological pathways enriched by intestinal microbiome genes associated with CRC progression, We used Phylogenetic Investigation of Communities by Reconstruction of Unobserved States 2 (PICRUSt2)—the software applied to predict KEGG pathways between the both groups for further analysis and a total of 180 KEGG pathways were identified (Additional file 10: Table S4), only two of them had significant statistical differences (Wilcoxon, P < 0.05). The abundance of ko00514: Other types of O − glycan biosynthesis in stage III-IV group was significantly higher than that in stage I-II group (Wilcoxon, P = 0.018). Conversely, the ko00590: Arachidonic acid metabolism pathway abundance of stage I-II group was significantly higher than that in the stage III-IV group (Wilcoxon, P = 0.012) (see Fig. 3 and Additional file 10: Table S4 for details). What showed above revealed the different metabolic functions of intestinal microbiome associated with CRC progression.

### Relationship between CRC progression-associated microbiota and tumor-infiltrating immune cells

**Fig. 4:**
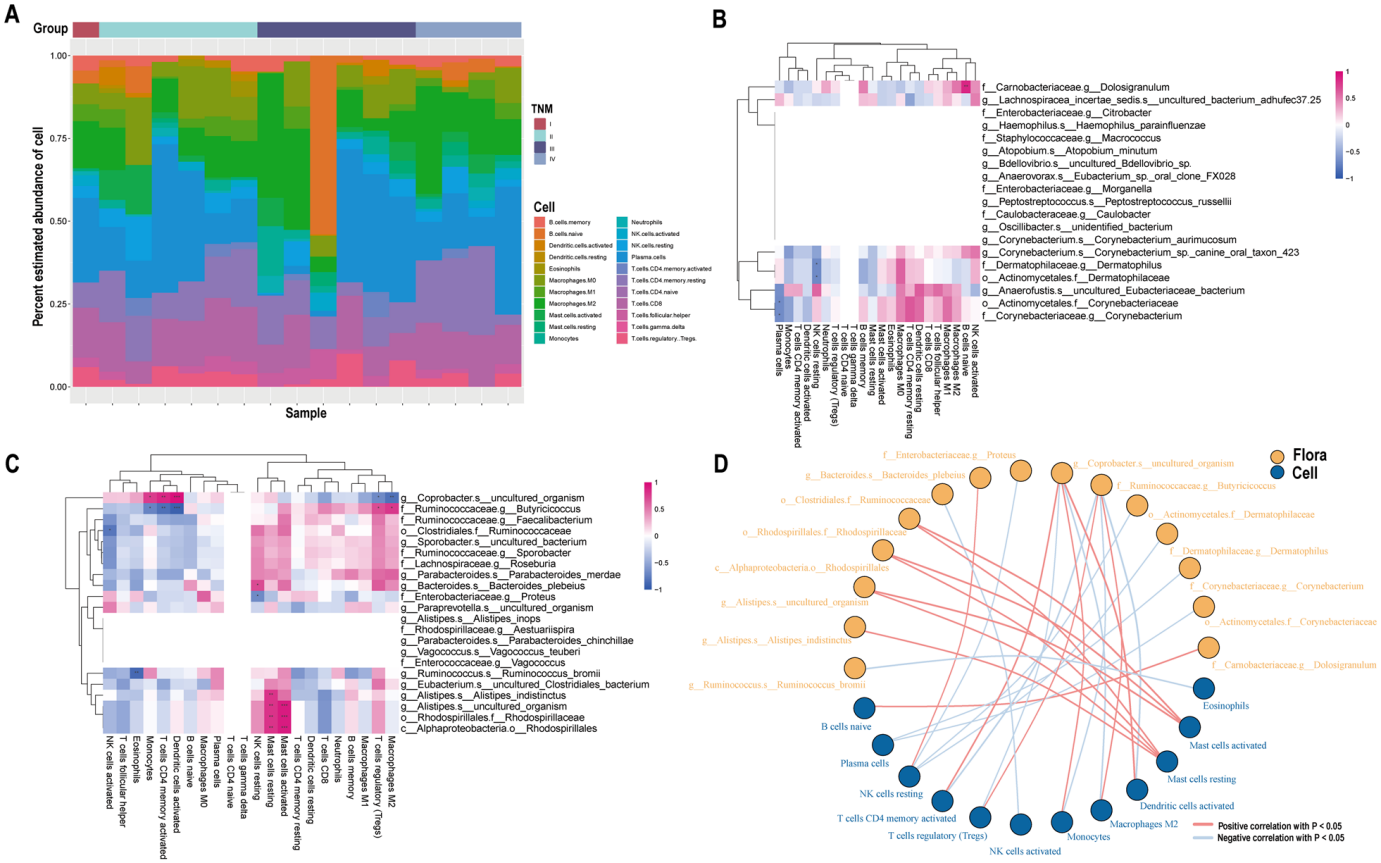
Correlation of CRC progression-related differential intestinal flora with tumor immune infiltrating cells. **A** Relative abundance strip chart of immune cells in CRC patients grouped by tumor stage. Each bar is a sample, and the vertical coordinate is the predicted relative abundance value of immune cells. The sum of the relative abundance of all immune cells in a single sample is 1, and each color in the graph corresponds to one immune cell. **B** Heat map of correlation between dominant flora and immune cell abundance in stage I-II group. **C** Heat map of correlation between dominant flora and immune cell abundance in stage III-IV group. The horizontal coordinates indicate immune cells. The vertical coordinates indicate bacteria. The red color in the graph represents positive correlation. The blue color represents negative correlation. The color depth represents the Pearson correlation coefficient size. The color from light to dark indicates the Pearson correlation coefficient value from small to large. The "*" in the graph indicates the size of P-value: no * for P-value ≥ 0.05, * for 0.01 ≤ P < 0.05, ** for 0.001 ≤ P < 0.01, *** for P < 0.001. **D** Network diagram of correlation between associated differential intestinal flora and immune cells. Each node represents each gut bacteria or immune cell. The node color represents the grouping. And the connecting line represents the existence of significant correlation between two nodes. The Spearman correlation coefficient value less than 0 (negative correlation) indicates the blue line, and the Spearman correlation coefficient value greater than 0 (positive correlation) indicates the red line

Tumor-infiltrating immune cells are important constituents of tumor microenvironment and participates in regulating anti-tumor immune response, which is a potential therapeutic target for tumor immunotherapy. To decipher the interplay between CRC progression-associated differential microbiota and tumor-infiltrating immune cells, we analyzed the data of 17 CRC patients with certain TNM stage and RNA sequencing and depicted the band map of immune cell abundances (Fig. 4A).From the band map, we observed different immune cell infiltration among different TNM stage of CRC patients. Then, in order to explor the relationship between CRC progression-associated differential bacteria and immune cells, we performed correlation analysis among these CRC progression-associated intestinal microbiome and 22 immune cells. In stage I-II CRC group, there was a significant positive correlation between *f__Carnobacteriaceae.g__Dolosigranulum* and B cells naive, *f__Dermatophilaceae.g__Dermatophilus* and *o__Actinomycetales.f__Dermatophilaceae* are negatively correlated with NK cell resting significantly (Fig. 4B, D). In stage III-IV CRC group, *g__Alistipes.s__uncultured_organism, o__Rhodospirillales.f__Rhodospirillaceae* and *c__Alphaproteobacteria.o__Rhodospirillales* are positively proportional to Mast cells resting and Mast cells activated dominantly (Fig. 4C, D). *f__Ruminococcaceae.g__Butyricicoccus* was markedly negatively correlated with Dendritic cells activated and T cells CD4 memory activated. In conclusion, the differential flora associated with CRC progression are significantly correlated with a variety of immune cells, suggesting their potential role of regulating immune infiltration in CRC.

### Correlation between CRC progression-associated differential microbiota and immune-related genes

**Fig. 5:**
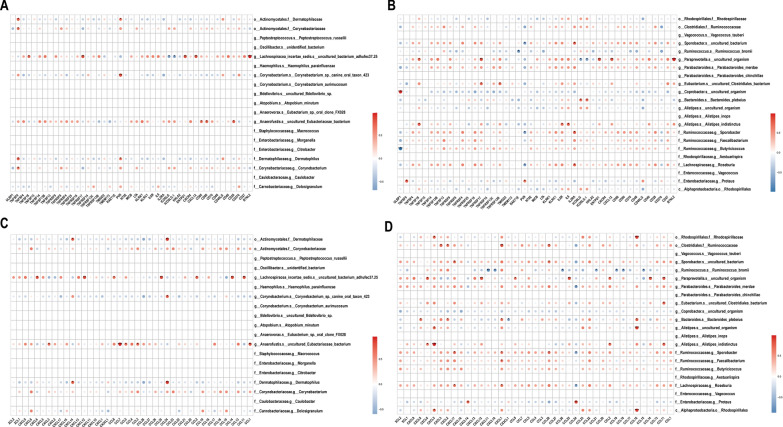
Correlation of CRC progression-related differential intestinal flora with immune-related genes. **A** Heatmap of the correlation between dominant flora and immune activation genes in stage I-II CRC. **B** Heatmap of the correlation between dominant flora in stage III-IV CRC and immune activation genes. **C** Heatmap of the correlation between the dominant flora in stage I-II CRC and chemokines. **D** Heatmap of the correlation between dominant flora in stage III-IV CRC and chemokines. The horizontal coordinates indicate genes. The vertical coordinates indicate colonies. Red in the graph represents positive correlation. Blue represents negative correlation. Color depth represents Pearson correlation coefficient size. Color from light to dark indicates Pearson correlation coefficient value from small to large. The "*" in the graph indicates the size of P-value: no * for P-value ≥ 0.05, * for 0.01 ≤ P < 0.05, ** for 0.001 ≤ P < 0.01, *** for P < 0.001

Tumor occurrence and development are closely associated with host immunity. To investigate the relationship between CRC progression -associated differential microbiota and host immunity, correlation analysis was performed among the differential bacteria and common immune-related genes. Among the dominant bacteria in stage I-II CRC group, *g__Lachnospiracea_incertae_sedis.s__uncultured_bacterium_adhufec37.25* had observable positive correlation with multiple checkpoints (TNFSF18, BTNL2 and VTCN1 etc.) (see Additional file 1: Fig. S1), immune-activating genes (TNFRSF17, ENTPD1 and CXCL12 etc.)(Fig. 5A), immunosuppressive genes (LAG3, KDR and IL10, etc.) (as in Additional file 2: Fig. S2), chemokines (CXCL6, CCL8 and CCL14 etc.) (as in Fig. 5C) and chemokine receptors (CCR2) (as in Additional file 3: Fig. S3). For the dominant bacteria in stage III-IV CRC,*g__paraprevotelela.s__uncultured_organism* was markedly relevant with multiple checkpoints (ADORA2A, VTCN1 and BTNL2 etc.) (as in Additional file 4: Fig. S4), immune-activating genes (ENTPD1, TNFSF18 and TNFRSF17 etc.) (as in Fig. 5B), immune-suppressive genes (KDR and LAG3 etc.) (as in Additional file 5: Fig. S5), chemokines (CCL11, ﻿CXCL12﻿ and CCL25 etc.) (Fig. 5D) and chemokine receptors (CCR2) (as in Additional file 6: Fig. S6 showed). Over all, the differential microbiota associated with CRC progression may affect the expression of immune-related genes.

### Identification of differential functional pathways and their correlation with CRC progression-associated differential microbiota

**Fig. 6:**
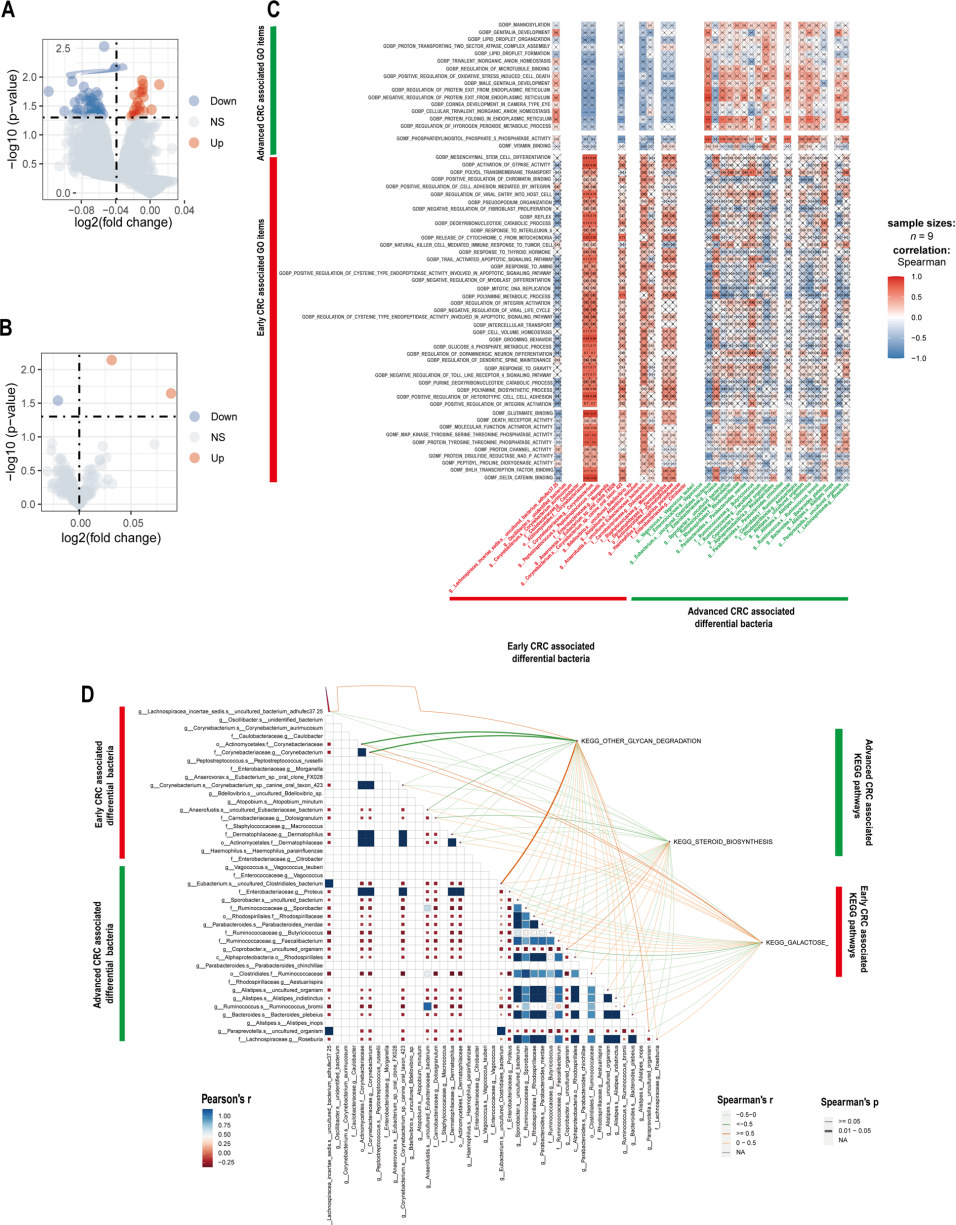
Identification of CRC progression-associated differential pathway and its correlation with CRC progression-associated differential intestinal flora. **A** GO volcano plot of the associated differential expression in the stage I-II CRC versus the stage III-IV CRC. **B** KEGG Volcano plot of the associated differential expression in stage I-II CRC vs. stage III-IV CRC. The horizontal coordinate indicates log2 (fold change). The farther the dot is from the center, the greater the fold of difference; the vertical coordinate indicates -log10 (p value). The closer to the top the point indicates, the more significant the difference in expression. Each dot represents a detected differentially expressed gene. Red indicates up-regulated genes. Blue indicates down-regulated genes. And gray indicates no differential genes. **C** Correlation graph of BP and MF of CRC progression-related differences with CRC progression-related differential intestinal microbiome. The horizontal coordinates indicate bacteria. The vertical coordinates indicate GO labels. The red color in the graph represents positive correlation. The blue color represents negative correlation. The color depth represents Spearman correlation coefficient size. The color from light to dark indicates Spearman correlation coefficient value from small to large. The " × " in the graph indicates the size of P-value: with × means P-value ≥ 0.05, without × means P < 0.05. **D** Correlation graph of CRC progression-related differences in KEGG and CRC progression-related differential intestinal flora. The triangle graph represents the correlation graph of CRC progression-related differential intestinal flora. The upper right corner is the KEGG label. Red font indicates the KEGG pathway upregulated in the stage III-IV group. Blue font indicates the KEGG pathway upregulated in the stage I-II group. The depth of the connecting color represents the magnitude of Spearman's correlation coefficient “r”. The connecting line presented in dark orange indicates the correlation coefficient r ≥ 0.05, representing a strong positive correlation. The connecting line presented in light orange indicates the correlation coefficient 0 < r < 0.05, representing a weak positive correlation. A dark orange line indicates a strong positive correlation; a light orange line indicates a correlation coefficient of 0 < r < 0.05, which represents a weak positive correlation. A dark green line indicates a correlation coefficient r ≥ 0.05, representing a strong negative correlation; a light green line indicates a correlation coefficient 0 < r < 0.05, representing a weak negative correlation; the thickness of the line represents the p-value, with a thick line representing 0.01 < P < 0.05 and a thin line representing P ≥ 0.05

To identify the differential regulatory pathways associated with CRC progression and the correlation among CRC progression-associated differential intestinal microbiome and these pathways, we transformed RNA sequencing data of cancer tissue samples from 9 CRC patients (their 16sRNA sequencing data of intestinal microbiome is also available) into the corresponding scoring matrix of Gene Ontology (GO) and Kyoto encyclopedia of Genes and Genomes (KEGG) pathways using ssGSEA method. GO items included three aspects: biological process (BP), cellular component (CC) and molecular function (MF). Next, through differential analysis of the GO and KEGG pathway score matrices between the stage I-II and stage III-IV CRC group, we found a total of 46 GO items [GOBP_POSITIVE_REGULATION_OF_INTEGRIN_ACTIVATION (logFC = − 0.053, P = 0.003) and GOBP_NEGATIVE_REGULATION_OF_TOLL_LIKE_RECEPTOR_4_SIGNALING_PATHWAY (logFC =  – 0.053,P = 0.009) etc.], and one KEGG pathway[KEGG_GALACTOSE_METABOLISM(logFC = − 0.021, P = 0.029)] were significantly up-regulated in the stage I-II CRC group. For group stage III-IV CRC, 19 GO items[GOBP_CELLULAR_TRIVALENT_INORGANIC_ANION_HOMEOSTASIS(logFC = – 0.050,P = 0.013) and GOBP_PROTEIN_FOLDING_IN_ENDOPLASMIC_RETICULUM (logFC = 0.031, P = 0.013) etc.], and 2 KEGG pathways [KEGG_OTHER_GLYCAN_DEGRADATIO (logFC = 0.031, P = 0.007) and KEGG_STEROID_BIOSYNTHESIS(logFC = 0.089, P = 0.023)] were significantly up-regulated (Fig. 6A, B). Detailed enriched GO item and KEGG pathway lists are shown in Additional file 10: Table S4, Additional file 11: Table S5, Additional file 12: Table S6 respectively. And the above results indicated that different biological functions are enriched in CRC of different stages.

Subsequently, in order to explore the relationship between CRC progression-related genomic functions and the dominant intestinal microbiome associated with CRC progression, we conducted the correlation analysis among 41 CRC progression-associated differential intestinal microbiome and score matrices of BP, MF and KEGG pathway that are related to CRC progression from 9 CRC patients. Remarkable correlation among some differential intestinal microbiome and BP, MF and KEGG pathways were found, such as GOBP_NEGA TIVE_REGULA TION_OF_PROTEIN_EXIT_FROM_ENDOPLASMIC_RETICULUM was strongly positively proportional to *g__Eubacterium.s__uncultured_Clostridiales_bacterium* (Pearson r = 0.89, P < 0.05)(Fig. 6C), and there was a significant negative correlation between KEGG_OTHER_GLYCAN_DEGRADATION and *f__Corynebacteriaceae.g__Corynebacterium* (Pearson r = − 0.68, P < 0.05)(Fig. 6D).In summary, CRC progression-associated differential intestinal microbiome may affect the progression of CRC through some potential biological functions.

### Intestinal microbiome signature construction for predicting the tumor stage of CRC based on RF and XGB prediction models

For the sake of selecting potential intestinal microbiome biomarkers associated with CRC progression and predicting the CRC stage more accurately, we constructed Random Forest (RF) and eXtreme Gradient Boosting (XGBoost) prediction models based on the 41 CRC progression -associated intestinal microbiome obtained by Linear discriminant analysis Effect Size (LEfSe) analysis.

**Fig. 7:**
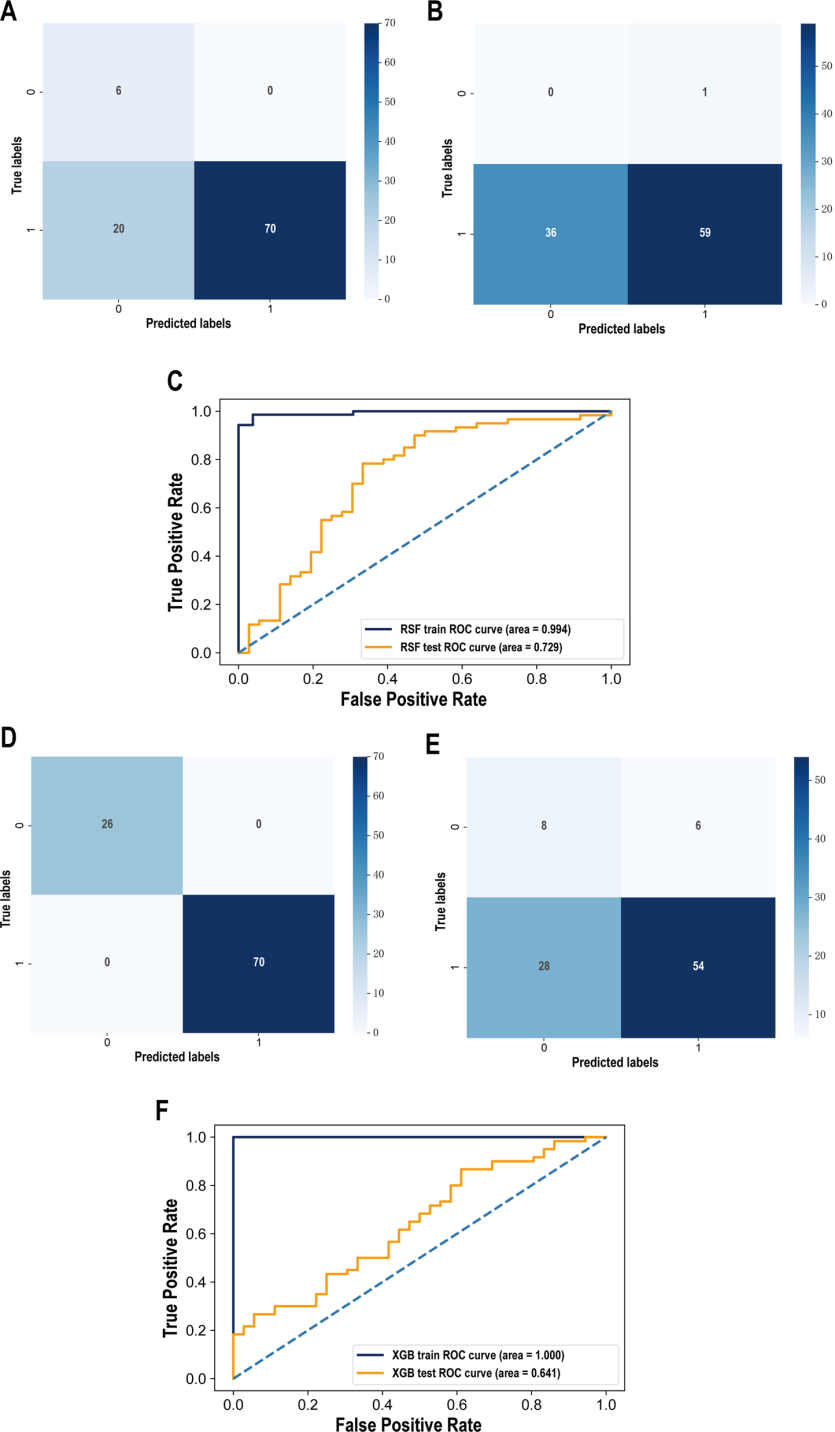
Evaluation of the effect of RF and XGB prediction models. **A** Confusion matrix of RF model in the training set. **B** Confusion matrix of the RF model for the validation set. C ROC curves of RF model in both training set and validation set. **D** Confusion matrix of the XGB model in the training set. **E** Confusion matrix of the XGB model in the validation set. F ROC curves of XGB model in both training set and validation set. The x-axis represents the predicted situation of the model. The y-axis represents the true situation. 1 represents correct prediction. 0 represents incorrect prediction. And the value in the box is the sample size. The horizontal coordinate is the false positive rate predicted by the model, the vertical coordinate indicates the true positive rate predicted by the model, and the area under the curve represents the AUC value; the higher the AUC value, the higher the diagnostic efficacy of the model

The training set confusion matrix of the RF-based CRC staging prediction model showed that the number of predicted true positive (TP) samples was more than that of false positive (FP) samples, but the number of predicted true negative (TN) samples was significantly less than that of false negative (FN) samples (Fig. 7A). The confusion matrix of the validation set was similar to the training set, whose number of TP samples was more than that of FP samples, but the number of TN samples was significantly less than that of FN samples (Fig. 7B). The AUC value of ROC curve was 0.994 in the training set and 0.729 in the validation set respectively (Fig. 7C). The RF-based CRC staging prediction model had an AUC value of greater than 0.7 in both the training and validation sets. The number of samples predicted as TN and TP by the confusion matrix of the training set was significantly more than that of FN and FP samples (Fig. 7D). For the XGBoost-based CRC staging prediction model, the confusion matrix of validation set showed that the number of samples predicted as TP was more than that of FP samples. However, the number of TN samples was significantly less than the number of FN samples (Fig. 7E). The AUC value of ROC curve in the training set was 1.000, whereas that in the validation set was 0.641 (Fig. 7F), which was less than 0.7. Although the confusion matrices of both models presented a high false negative rate, both models had certain prediction accuracy, and the RF-based CRC staging prediction model had higher prediction efficiency than XGBoost-based model, which could contribute to the practice of CRC staging prediction by intestinal microbiome detection.

## Discussion

Based on 16S rRNA gene sequencing of fecal samples, this study explored the differences of intestinal microbial communities between patients with colorectal cancer (CRC) at stages I-II and III-IV, identifying significantly enriched microbiota in feces of CRC patients with stage I-II and stage III-IV respectively. We compared the intestinal microbiome richness and diversity between stage I-II CRC and stage III-IV CRC patients. Simpson index was statistically remarkably lower in CRC patients with stage III-IV than those with stage I-II (Fig. 1A, Wilcoxon, P = 0.043), which indicated that the diversity and evenness of intestinal microbiome in stage III-IV CRC patients were significantly higher than that of stage I-II CRC patients. Although there was no significant difference in β diversity index between the two groups (Fig. 1B, C), the intestinal microbiome could be obviously divided into two groups after Partial Least squares-discriminant Analysis (PLS-DA) (Fig. 1C), which pointed out that intestinal microbiome compositions of CRC patients with stage I-II and stage III-IV were different to distinguish. One study had shown that the observed OTUs and Chao1 index of intestinal microbiome in CRC patients was significantly higher than that of healthy controls [16]. And another research indicated that the Simpson index of fecal flora in CRC patients was significantly lower than that of health volunteers [17]. Some researchers also compared the diversity of intestinal microbiome in CRC with different TNM stages, suggesting that the Simpson index of CRC patients with stage IV was significantly lower than that of stage III [18]. Therefore, we reasoned that the abundance and diversity of intestinal microbiome may gradually increase with the development and progression of CRC.

We selected differential intestinal microbiome associated with CRC progression based on Linear discriminant analysis Effect Size (LEfSe). At the genus level, *Proteus*, *Parabacteroides*, *Alistipes* and *Ruminococcus* were in significant enrichment in the fecal microbiota of stage III-IV CRC patients. *Proteus* is a pathogenic Gram-negative bacterium widely distributed in soil and water sources [19], which has recently been found to be closely associated with CRC. L Wachsmannova etc. found *Proteus* mirabilis presented inside the cells of colorectal adenomas and CRC, whereas its presence was not detected in normal colorectal mucosal cells [20]. After co-culture with *Proteus vulgaris* and *Proteus mirabilis*, SNAI1 and VIM gene expressions had changed significantly in human colon cancer cell line Caco2, which are related to epithelial mesenchymal transformation, thus it was suggested that *Proteus* may be correlated with the epithelial mesenchymal transformation of CRC [21]. Moreover, increased abundance of *Proteus mirabilis* played a key role in the liver metastasis of CRC, which may count on the reduction of Kupffer cells in the liver [22]. *Parabacteroides* was observed to be distinctly higher in abundance in CRC tissues than normal mucosa adjacent to cancer [23], besides, CRC patients with high *Parabacteroides* expression in gut had observably poorer prognosis than CRC patients with low *Parabacteroides* expression in gut [24]. As a CRC-related pathogenic bacterium, *Alistipes* is associated with depressive mental state [25], Multiple studies have shown that compared to healthy controls, *Alistipes* genus was dominantly enriched in CRC mucosa [26–28], Jihye Park etc. found that the *Alistipes* abundance of CRC patients with stage IV was remarkably declined than that of CRC patients with stage I-III [26], which was consistent with the variation trend of *Alistipes* abundance in our study. *Ruminococcus* genus colonizes in the large intestine of human body [29], capable of secreting pro-inflammatory substances which could cause inflammatory bowel disease [30, 31]. In recent years, it is considered to be closely related to CRC [32]. Previous researches had shown that, either in the fetes sample [33–35] or on the CRC mucosa [36], in comparison with the normal control, the abundance of *Ruminococcus* was distinctly elevated. In conclusion, we speculated that *Proteus*, *Parabacteroides*, *Alistipes* and *Ruminococcus* were strongly bound up with CRC progression, accompanying with increased abundance of which might boost the development and progression of CRC.At the species level, *Bacteroides plebeius* is shown to be closely linked to colorectal cancer. Compared with healthy people, the abundance of *Bacteroides plebeius* in the fecal microbial community of patients with adenomatous polyps, CRC and Lynch syndrome was dominantly elevated [37, 38]. Combined with other bacteria, *Bacteroides plebeius* can be used as a highly effective biomarker to distinguish colorectal adenoma from CRC [39]. It is worth noting that in our study, the fecal abundance of *Bacteroides plebeius* of stage III-IV CRC patients was significantly higher than that of stage I-II patients, exhibiting an upward trend with the progress of CRC. Although Hanju Hua et c. observed a remarkable decline in *Bacteroides* genus abundance of CRC patients compared to patients with adenomatous polyps, there was no clear evidence that the abundance of *Bacteroides plebeius* descended observably in the fecal microbiota of CRC patients [37]. Therefore, we hypothesised that *Bacteroides plebeius* is relevant to the development of CRC, increased abundance of which may be a biological landmark event along the adenoma-carcinoma sequence, but it remains to be further explored.

In Comparison with stage III-IV CRC patients, *Anaerovorax* and *Oscillibacter* were dominantly abundant in fecal microbia of stage I-II CRC patients, both of which were confirmed to be probiotics [40, 41]. *Anaerovorax* is an anaerobic Gram-positive bacterium, which participates in short chain fatty acids (SCFA) such as acetate and butyrate production [42]. SCFA can not only be served as the main energy source of intestinal mucosal cells, but also as an anti-inflammatory compound, reducing the production of intestinal pro-inflammatory factors [43]. *Oscillibacter*a also takes a role in SCFA production [44], by which *Oscillibacter*a reduces Th17 cell polarization and boost the differentiation of intestinal anti-inflammatory cells Treg and Trl cells [45]. Furthermore, it was reported that *Oscillibacter* induced a decline in blood triglycerides [46], and high levels of triglycerides are a risk factor for CRC [47, 48]. *Oscillibacter* abundance in gastrointestinal tract neoplasms was remarkably reduced compared to the healthy controls [49]. In addition, We observed that the fecal abundance of *Atopobium minutum* in CRC patients with stage I-II was significantly higher than that in CRC patients with stage III to IV. *Atopobium minutum* was confirmed to be capable of inducing apoptosis of CRC cells, presenting its potential probiotic role [50]. Although the previous researches and our results manifested that *Anaerovorax*, *Oscillibacter* and *Atopobium minutum* are all probiotics, the addition of a healthy control group will be more conducive to exploring their relationship with the development and progression of CRC.

In the next place, we operated the Phylogenetic Investigation of Communities by Reconstruction of Unobserved States 2 (PICRUSt2) software for predicting the differential KEGG pathways of intestinal microbiome between stage III-IV and stage I-II CRC. in contrast to stage I-II CRC, ko00514: Other types of O − glycan biosynthesis were in significant enrichment in stage III-IV CRC (Fig. 3). O − glycans are glycoproteins connecting carbohydrate chains in the form of O-glycosides. They are widely distributed in cell membranes and secretions of cancer cells with complex structure and biological functions [51], which contribute to tumor progression and can be served as potential biomarkers for carcinogenesis and staging prediction [52]. Several studies have confirmed that O-glycan inhibitors can increase content of intracellular aryl-glycans by blocking the activity of glycosyltransferase inside the tumor cells, so as to induce tumor apoptosis and suppress tumor proliferation [53, 54]. Therefore, we speculated that CRC with stage III-IV might stimulate its invasion and proliferation by enhancing the synthesis of O − glycans, thus achieving the progression of CRC.

Subsequently, by exploring the relationship among the CRC progression-associated differential intestinal microbiome, immune infiltrating cells and immune-related genes, we did find that *Alistipes* genus had a remarkable positive correlation with mast cells (Fig. 4C), amidst which, *Alistipes indistinctus* manifested observable positive correlation with immune activators IL-6 and IL-6R. several studies had shown that inflammation and tumorigenesis in colon were accompanied by increased *Alistipes* abundance and elevated IL-6 level in the body [55–57], which implied that there may be a positive correlation between *Alistipes* and IL-6, which is consistent with our analysis. IL-6 can be produced by a variety of cells in different disease states [58], proved to be a key cytokine in promoting mast cell proliferation and maturation [59, 60]. In the presence of stem cell factor, IL-6 directly boosted the differentiation and development of CD34^+^ cells in cord blood into mast cells through the IL-6R-gp130 system [61]. Studies have documented that mast cells massively infiltrate in the tumor microenvironment of CRC, releasing proangiogenic factors and metalloproteinases to promote the angiogenesis and invasion of CRC respectively [62]. Thus, we hypothesized that *Alistipes indistinctus* induced the proliferation and maturation of infiltrated mast cells by stimulating the production of IL-6, thus facilitating the progression of CRC, but it still needs to be validated by further research.

Furthermore, we observed *Alistipes indistinctus* was strongly positively proportional to GOBP_PROTEIN_FOLDING_IN_ENDOPLASMIC_RETICULUM, which was dominantly upregulated in stage III-IV CRC (Fig. 6C, Spearman r = 0.71, P < 0.05). Endoplasmic reticulum (ER) is the main organelle responsible for protein synthesis, folding and secretion. Due to its limited ability to process proteins, unfolded or misfolded proteins will accumulate excessively in the ER lumen when in pathological state, leading to ER stress [63]. ER stress may trigger the unfolded protein response (UPR), which is a signal network that coordinating function of endoplasmic reticulum for homeostasis restoration, and be able to reduce the load of unfolded proteins and maintain the normal vitality and function [64]. ER stress and UPR had been shown to be closely associated with inflammatory bowel disease and CRC [65], Yugang Wang etc. revealed that high expression of PERK contributed to increased tumor vascular density and tumor growth by activating the UPR, which promoted the production of pro-angiogenic mediators and reduced the production of pro-angiogenic inhibitors [66]. It is noteworthy that a research observed both elevated abundance of *Alistipes* in intestinal microbiome and myocardial PERK expression in diabetic cardiomyopathy models of mice, and after drug intervention, the expression levels of both declined [67], so it is highly likely that there is a positive correlation between *Alistipes* abundance and PERK expression. Therefore, our speculation is that *Alistipes indistinctus* is conductive to the correct folding of proteins in the ER of CRC cells, thereby reducing ER stress and facilitating the survival and progression of CRC, which may be on account of high expression of PERK and activation of downstream UPR boosted by *Alistipes indistinctus*. Intriguingly, IL-6 is also known as a common proangiogenic factor [68],and it can also be produced by mast cell [59], Which raise a question: whether mast cells are involved in angiogenesis through activated downstream UPR of PERK and whether *Alistipes indistinctus* plays a role in the biological process need to be studied further.

Last but not the least, in order to explore the potential gut bacteria as fecal biomarkers to distinguish patients with stage I-II and stage III-IV CRC, this study included 42 characteristic bacteria related to CRC progression and constructed a random forest (RF) model and eXtreme Gradient Boosting (XGBoost) model, and compared the discrimination efficiency of the two models. Our results showed that the AUC value of the RF model for predicting CRC progression was greater than 70% in both the training set and the validation set (Fig. 7C). The AUC value of the ROC curve of the XGB-based prediction model in the training set was as high as 1, but its AUC value in the validation set was less than 0.7 (Fig. 7E), indicating that both models still had certain predictive ability, and the model prediction ability of the RF model was better than that of the XGB model, although the false negative rates of the two models were both high (Fig. 7A, B, D, E). These results suggest that the construction of machine learning models can better reveal the association between intestinal microbiome and CRC progression, and the CRC progression-associated bacteria identified in this study can be used as potential fecal biomarkers to distinguish patients with stage I-II CRC and stage III-IV CRC.

However, this study has some limitations. First of all, compared to 16S rRNA gene sequencing, shotgun metagenomic sequencing can be sequenced at greater depth, identify more species of bacteria, and provide comprehensive microbial gene content data that directly describes the biological function of specific genes rather than using a whole population of genes for functional prediction [69]. In recent years, the application of shallow whole-metagenome shotgun sequencing has attracted attention. Compared with traditional deep whole-metagenome shotgun sequencing (WMS), it is comparable in accuracy and affordable. It can be widely used in future clinical metagenomics research [70, 71]. Secondly, metabolomics can be used to detect the dynamic changes of metabolites of intestinal microbiome, which clearly shows the metabolic state of intestinal microbiome in the host, so it can more intuitively explore the relationship between intestinal microbiome and CRC occurrence and development from the molecular level.

Secondly, this study is a single-center, cross-sectional study, and the addition of multi-center data is helpful to enhance the persuasive and popularization of the conclusion. In addition, the lack of healthy controls in this study is not conducive to judge the relationship between stage I-II enriched bacteria and CRC progression. However, we aimed to analyze the differential intestinal microbiome associated with CRC progression. By comparing the difference of intestinal microbiome between patients with stage III-IV and stage I-II CRC, we can filter intestinal microbiome with the same cancer background, which is more conducive to screen out bacteria closely related to CRC progression. It is worth mentioning that the fecal microbiota only partially reflects the microbiota of the mucus layer of the intestinal mucosa. Combining the microbial communities of oral mucosa and intestinal mucosa will provide a deeper understanding of the composition and evolution of the entire gastrointestinal microbial system [71].

At last, this study only provides a superficial measurement of the abundance of gut bacteria associated with CRC progression, and further experimental studies will reveal the relationship between these characteristic flora and CRC progression.

## Conclusion

Conclusively, the present study revealed that the abundance and diversity of the intestinal microbiome of CRC patients may increase progressively with the onset and progression of CRC. Elevated abundance of *Proteus*, *Parabacteroides*, *Alistipes* and *Ruminococcus* may contribute to CRC progression. The KEGG pathway of ko00514: Other types O-glycan biosynthesis may be associated with CRC progression, and CRC progression may occur through enhanced synthesis of O-glycans. *Alistipes indistinctus* may facilitate mast cell development and maturation by stimulation of IL-6 production. *Alistipes indistinctus* may help in the correct folding of proteins in endoplasmic reticulum for the sake of CRC cells and promote further progression by alleviating ER stress, which may be correlated to the increased PERK expression and activation of its downstream UPR by *Alistipes indistinctus*, but it still requires further experimental exploration. Both the RF model and XGBoost model constructed with 42 CRC progression-associated intestinal microbiome had discriminative efficacy in distinguishing stage I-II between III-IV CRC patients. The CRC progression-associated intestinal microbiome identified in this study could be used as promising microbial markers, which will be beneficial for the guidance of CRC staging diagnosis.

## Supplementary Information

**Additional file 1: Figure S1.** Heatmap of correlation between dominant flora and checkpoints in stage I-II CRC. The horizontal coordinates indicate genes. The vertical coordinates indicate colonies. Red in the graph represents positive correlation. Blue represents negative correlation. Color depth represents Pearson correlation coefficient size. Color from light to dark indicates Pearson correlation coefficient value from small to large. The "*" in the graph indicates the size of P-value: no * for P-value ≥ 0.05, * for 0.01 ≤ P < 0.05, ** for 0.001 ≤ P < 0.01, *** for P < 0.001.

**Additional file 2: Figure S2.** Heatmap of the correlation between dominant flora and immunosuppressive genes in stage I-IICRC. The horizontal coordinates indicate genes. The vertical coordinates indicate colonies. Red in the graph represents positive correlation. Blue represents negative correlation. Color depth represents Pearson correlation coefficient size. Color from light to dark indicates Pearson correlation coefficient value from small to large. The "*" in the graph indicates the size of P-value: no * for P-value ≥ 0.05, * for 0.01 ≤ P < 0.05, ** for 0.001 ≤ P < 0.01, *** for P < 0.001.

**Additional file 3: Figure S3.** Heatmap of the correlation between the dominant flora and chemokine receptors in stage I-II CRC. The horizontal coordinates indicate genes. The vertical coordinates indicate colonies. Red in the graph represents positive correlation. Blue represents negative correlation. Color depth represents Pearson correlation coefficient size. Color from light to dark indicates Pearson correlation coefficient value from small to large. The "*" in the graph indicates the size of P-value: no * for P-value ≥ 0.05, * for 0.01 ≤ P < 0.05, ** for 0.001 ≤ P < 0.01, *** for P < 0.001.

**Additional file 4: Figure S4.** Heatmap of correlation between dominant flora and checkpoints in stage III-IV CRC. The horizontal coordinates indicate genes. The vertical coordinates indicate colonies. Red in the graph represents positive correlation. Blue represents negative correlation. Color depth represents Pearson correlation coefficient size. Color from light to dark indicates Pearson correlation coefficient value from small to large. The "*" in the graph indicates the size of P-value: no * for P-value ≥ 0.05, * for 0.01 ≤ P < 0.05, ** for 0.001 ≤ P < 0.01, *** for P < 0.001.

**Additional file 5: Figure S5.** Heatmap of the correlation between dominant flora and immunosuppressive genes in stage III-IV CRC. The horizontal coordinates indicate genes. The vertical coordinates indicate colonies. Red in the graph represents positive correlation. Blue represents negative correlation. Color depth represents Pearson correlation coefficient size. Color from light to dark indicates Pearson correlation coefficient value from small to large. The "*" in the graph indicates the size of P-value: no * for P-value ≥ 0.05, * for 0.01 ≤ P < 0.05, ** for 0.001 ≤ P < 0.01, *** for P < 0.001.

**Additional file 6: Figure S6.** Heatmap of the correlation between the dominant flora and chemokine receptors in stage III-IV group. The horizontal coordinates indicate genes. The vertical coordinates indicate colonies. Red in the graph represents positive correlation. Blue represents negative correlation. Color depth represents Pearson correlation coefficient size. Color from light to dark indicates Pearson correlation coefficient value from small to large. The "*" in the graph indicates the size of P-value: no * for P-value ≥ 0.05, * for 0.01 ≤ P < 0.05, ** for 0.001 ≤ P < 0.01, *** for P < 0.001.

**Additional file 7: Table S1.** ADONIS test for Bray Distance of intestinal flora in patients with early and advanced CRC. Df: degrees of freedom, between-group degrees of freedom are the number of groups minus one, within-group degrees of freedom are the total number of samples minus the number of groups; Group row: between-group statistics; Residuals row: within-group statistics; Total row: between-group plus within-group statistics; Sums Of Sqs: sums of squares of deviations; Mean Sqs: mean square, ratio of sums of squares of deviations to degrees of freedom, i.e., Sums Of Sqs/Df; F.Model: F-test value, i.e., ratio of between-group mean square to within-group mean square; R2: Ratio of between-group and within-group's sums of squares of deviations to the sums of squares of the total deviations, indicating the degree of explanation of the difference between samples, and a larger R2 indicates a higher degree of explanation of the difference between samples; Pr(> F): the statistically significant P value obtained from the substitution test, and Pr < 0.05 is considered statistically significant.

**Additional file 8: Table S2.** ADONIS test for Jaccard Distance of intestinal flora in patients with early and advanced CRC. Df: degrees of freedom, between-group degrees of freedom are the number of groups minus one, within-group degrees of freedom are the total number of samples minus the number of groups; Group row: between-group statistics; Residuals row: within-group statistics; Total row: between-group plus within-group statistics; Sums Of Sqs: sums of squares of deviations; Mean Sqs: mean square, ratio of sums of squares of deviations to degrees of freedom, i.e., Sums Of Sqs/Df; F.Model: F-test value, i.e., ratio of between-group mean square to within-group mean square; R2: Ratio of between-group and within-group's sums of squares of deviations to the sums of squares of the total deviations, indicating the degree of explanation of the difference between samples, and a larger R2 indicates a higher degree of explanation of the difference between samples; Pr(> F): the statistically significant P value obtained from the substitution test, and Pr < 0.05 is considered statistically significants.

**Additional file 9: Table S3.** Results of LEfSe analysis. Taxonomy: information of differential species; LDA (log10): effect value of differential species; Group: group with significant abundance of differential species; the table shows species with LDA score (log10) P value less than 0.05 and greater than the present value (default is 2).

**Additional file 10: Table S4.** KEGG functional pathways in the intestinal microbiome of patients with early and advanced CRC. KEGG_Pathway: KEGG pathway; Mean In advanced stage: predicted abundance value of this pathway in each sample in stage III-IV group; Mean In early stage: predicted abundance value of this pathway in each sample in stage I-II group. P value < 0.05 is considered as statistically significant difference.

**Additional file 11: Table S5.** List of differential GO items of CRC patients stratified by CRC progression. GO items: Enriched GO entries. FC in logFC, i.e., fold change, indicates the ratio of expression in stage III-IV CRC group to that in stage I-II CRC group and is taken as logarithm with a base of 2. P.value < 0.05 is taken as statistically significant difference.

**Additional file 12: Table S6.** List of differential KEGG pathways of CRC patients stratified by CRC progression. KEGG pathway: enriched KEGG pathway. FC in logFC, fold change, i.e., indicates the ratio of expression in stage III-IV CRC group to  that in stage I-II CRC group and is taken as logarithm with a base of 2. P.value < 0.05 is taken as statistically significant difference.

## Data Availability

The original contributions presented in the study are included in the article material, further inquiries can be directed to the corresponding authors.
