# Supplementary figures and images for: Identification of colorectal cancer progression-associated intestinal microbiome and predictive signature construction
Source: J Transl Med. 2023 Jun 8;21:373. doi: 10.1186/s12967-023-04119-1 (PMC10249256; doi:10.1186/s12967-023-04119-1)

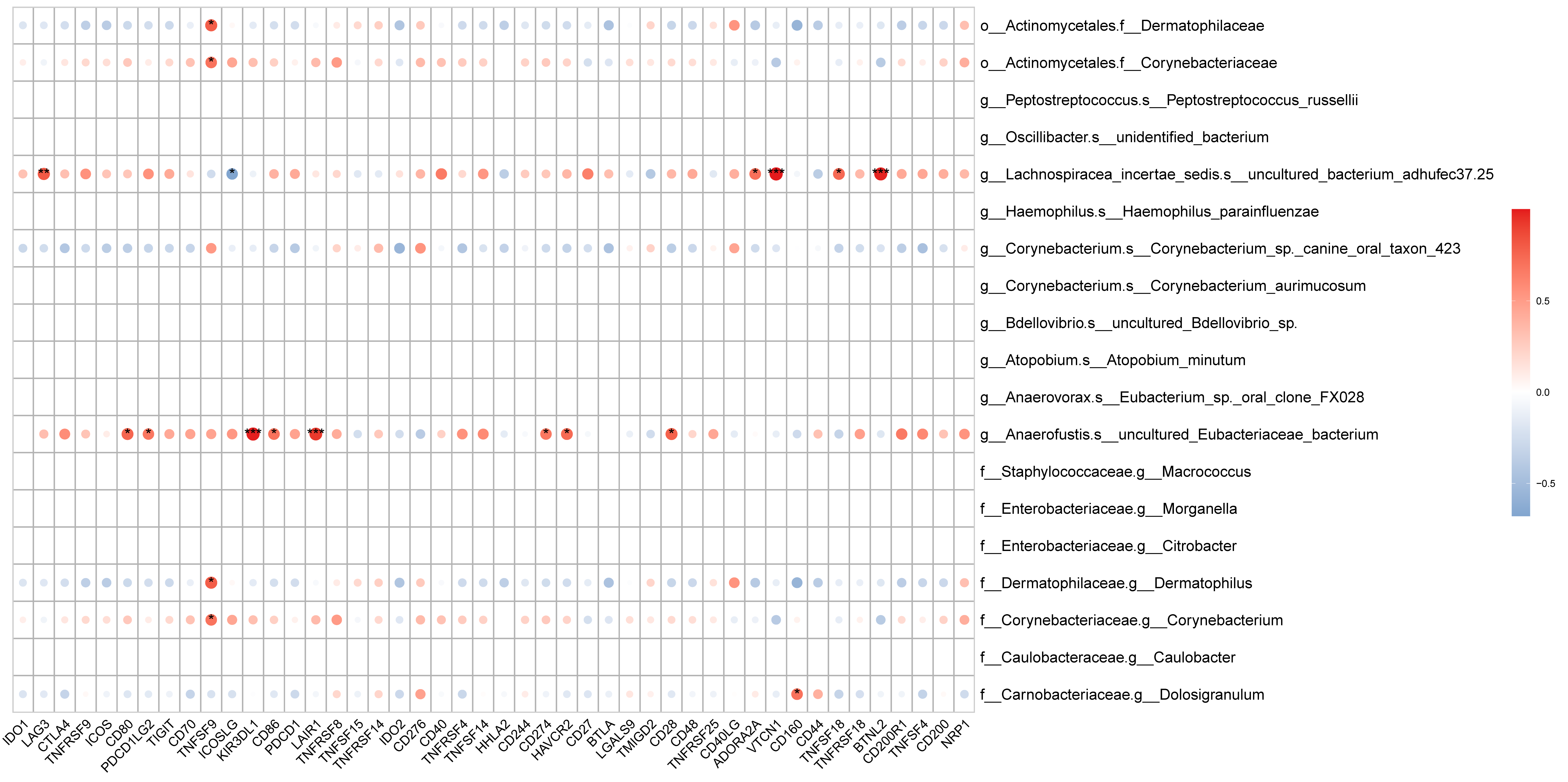

Supplement: Supplementary file 1 — Additional file 1: Figure S1. Heatmap of correlation between dominant flora and checkpoints in stage I-II CRC. The horizontal coordinates indicate genes. The vertical coordinates indicate colonies. Red in the graph represents positive correlation. Blue represents negative correlation. Color depth represents Pearson correlation coefficient size. Color from light to dark indicates Pearson correlation coefficient value from small to large. The "*" in the graph indicates the size of P-value: no * for P-value ≥ 0.05, * for 0.01 ≤ P < 0.05, ** for 0.001 ≤ P < 0.01, *** for P < 0.001. [file 12967_2023_4119_MOESM1_ESM.tif]

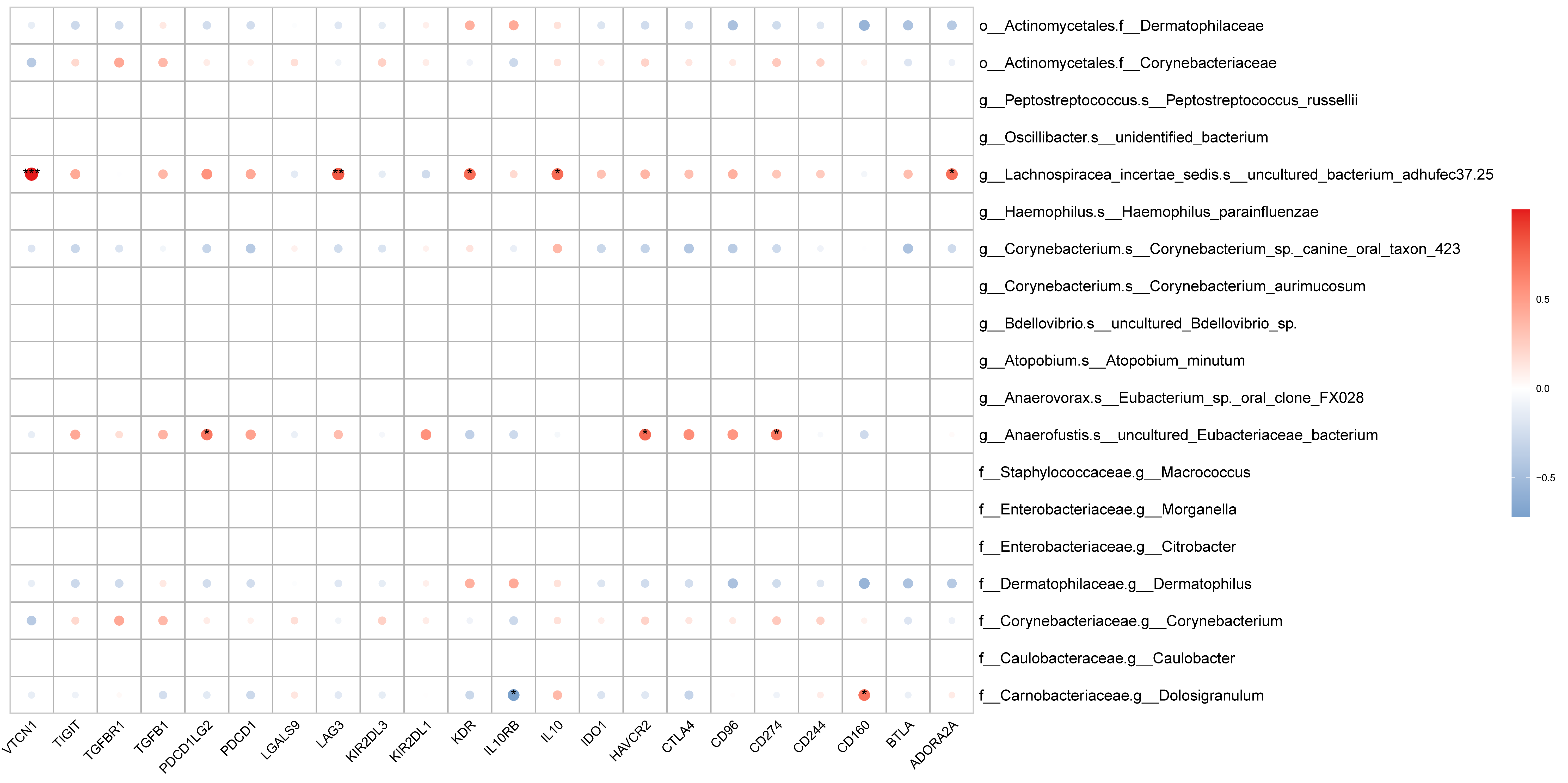

Supplement: Supplementary file 2 — Additional file 2: Figure S2. Heatmap of the correlation between dominant flora and immunosuppressive genes in stage I-IICRC. The horizontal coordinates indicate genes. The vertical coordinates indicate colonies. Red in the graph represents positive correlation. Blue represents negative correlation. Color depth represents Pearson correlation coefficient size. Color from light to dark indicates Pearson correlation coefficient value from small to large. The "*" in the graph indicates the size of P-value: no * for P-value ≥ 0.05, * for 0.01 ≤ P < 0.05, ** for 0.001 ≤ P < 0.01, *** for P < 0.001. [file 12967_2023_4119_MOESM2_ESM.tif]

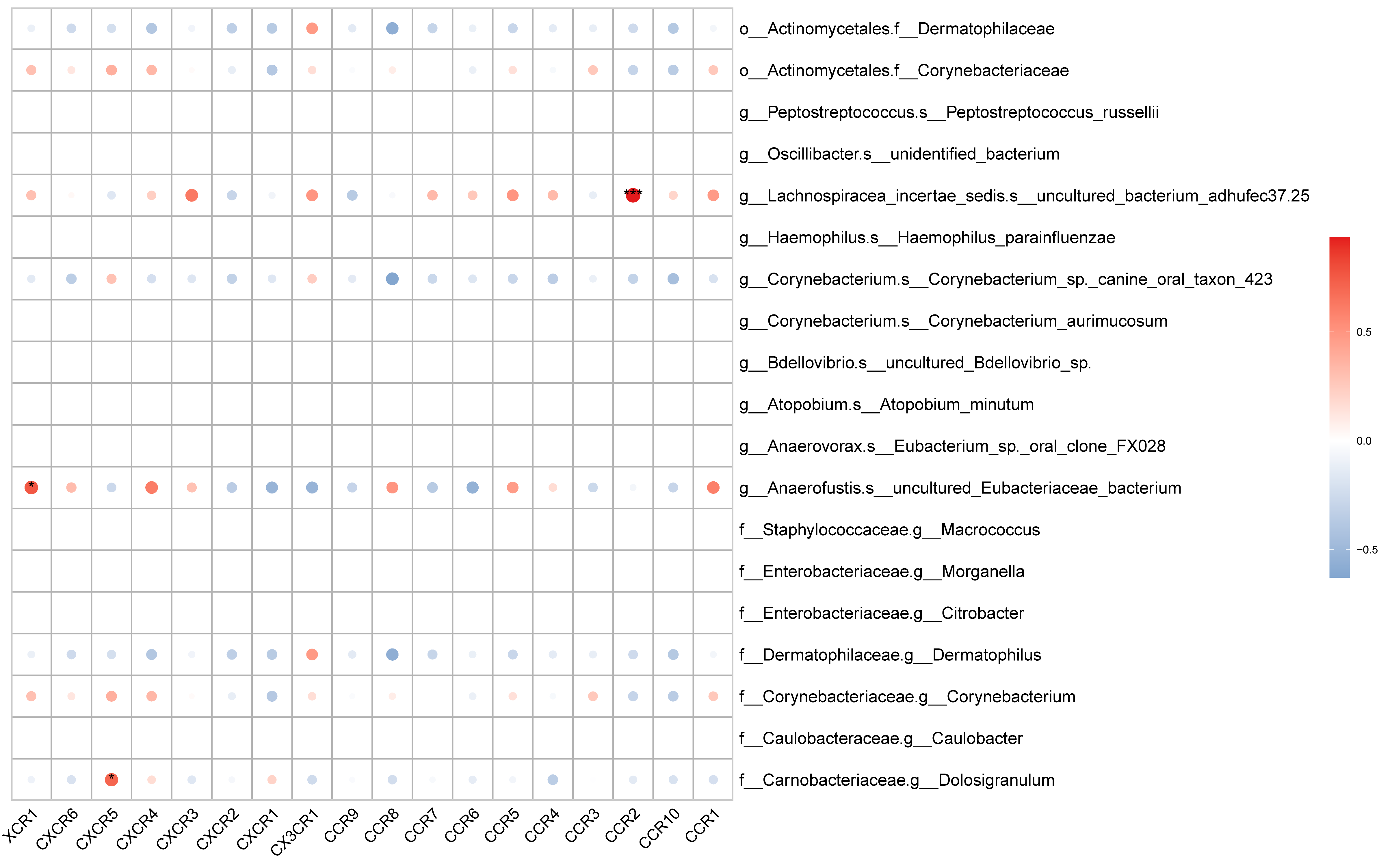

Supplement: Supplementary file 3 — Additional file 3: Figure S3. Heatmap of the correlation between the dominant flora and chemokine receptors in stage I-II CRC. The horizontal coordinates indicate genes. The vertical coordinates indicate colonies. Red in the graph represents positive correlation. Blue represents negative correlation. Color depth represents Pearson correlation coefficient size. Color from light to dark indicates Pearson correlation coefficient value from small to large. The "*" in the graph indicates the size of P-value: no * for P-value ≥ 0.05, * for 0.01 ≤ P < 0.05, ** for 0.001 ≤ P < 0.01, *** for P < 0.001. [file 12967_2023_4119_MOESM3_ESM.tif]

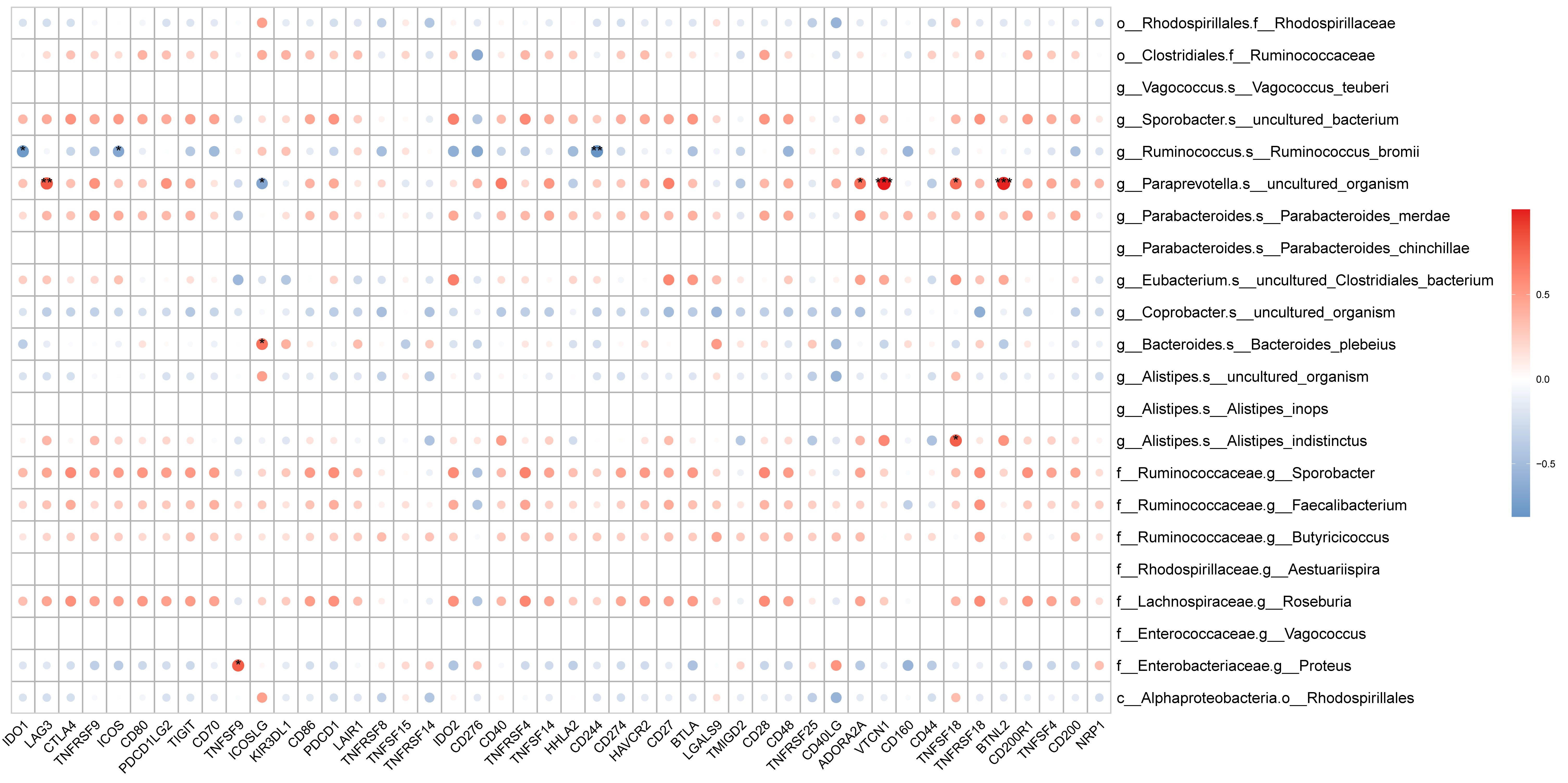

Supplement: Supplementary file 4 — Additional file 4: Figure S4. Heatmap of correlation between dominant flora and checkpoints in stage III-IV CRC. The horizontal coordinates indicate genes. The vertical coordinates indicate colonies. Red in the graph represents positive correlation. Blue represents negative correlation. Color depth represents Pearson correlation coefficient size. Color from light to dark indicates Pearson correlation coefficient value from small to large. The "*" in the graph indicates the size of P-value: no * for P-value ≥ 0.05, * for 0.01 ≤ P < 0.05, ** for 0.001 ≤ P < 0.01, *** for P < 0.001. [file 12967_2023_4119_MOESM4_ESM.tif]

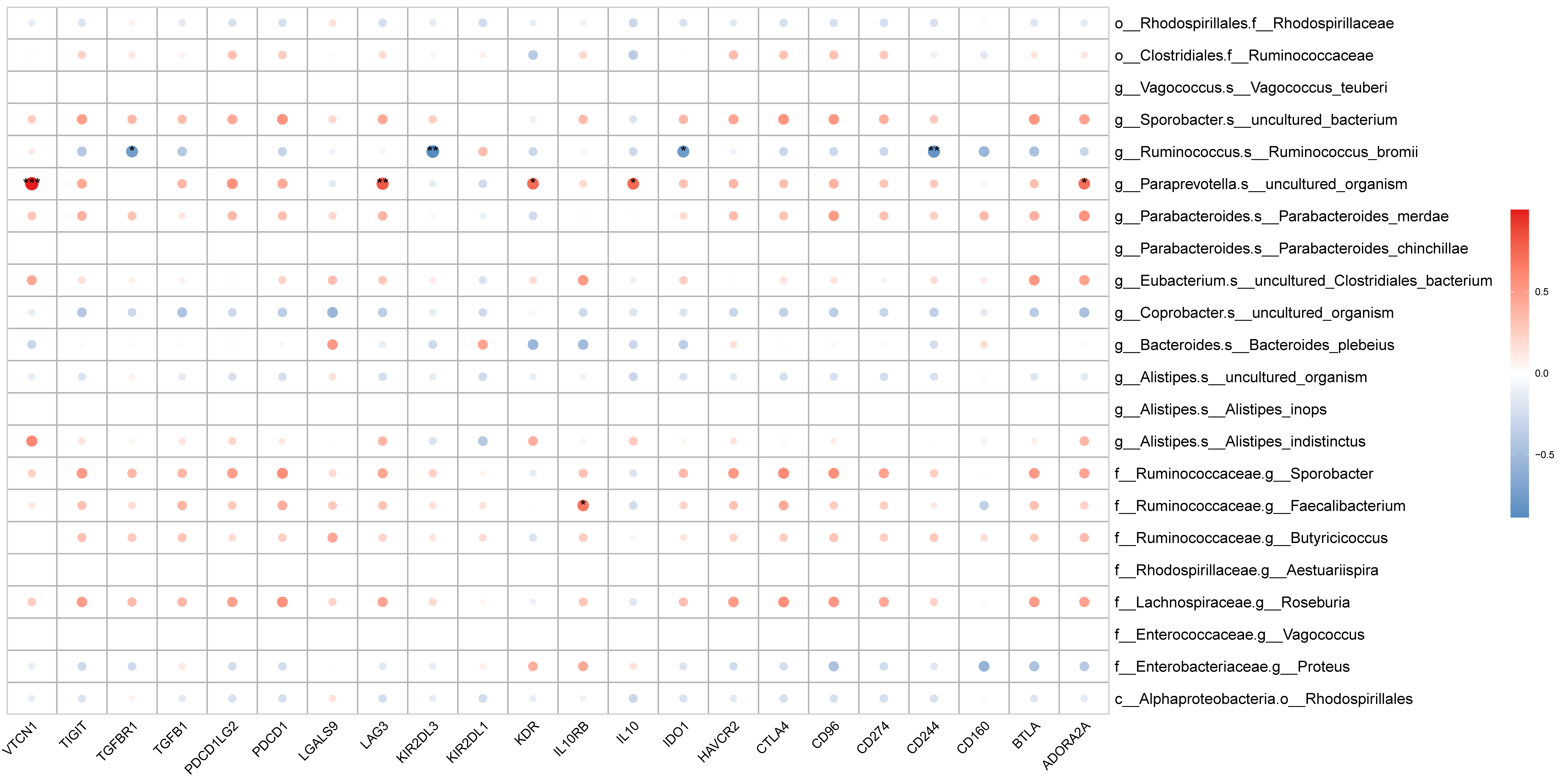

Supplement: Supplementary file 5 — Additional file 5: Figure S5. Heatmap of the correlation between dominant flora and immunosuppressive genes in stage III-IV CRC. The horizontal coordinates indicate genes. The vertical coordinates indicate colonies. Red in the graph represents positive correlation. Blue represents negative correlation. Color depth represents Pearson correlation coefficient size. Color from light to dark indicates Pearson correlation coefficient value from small to large. The "*" in the graph indicates the size of P-value: no * for P-value ≥ 0.05, * for 0.01 ≤ P < 0.05, ** for 0.001 ≤ P < 0.01, *** for P < 0.001. [file 12967_2023_4119_MOESM5_ESM.tif]

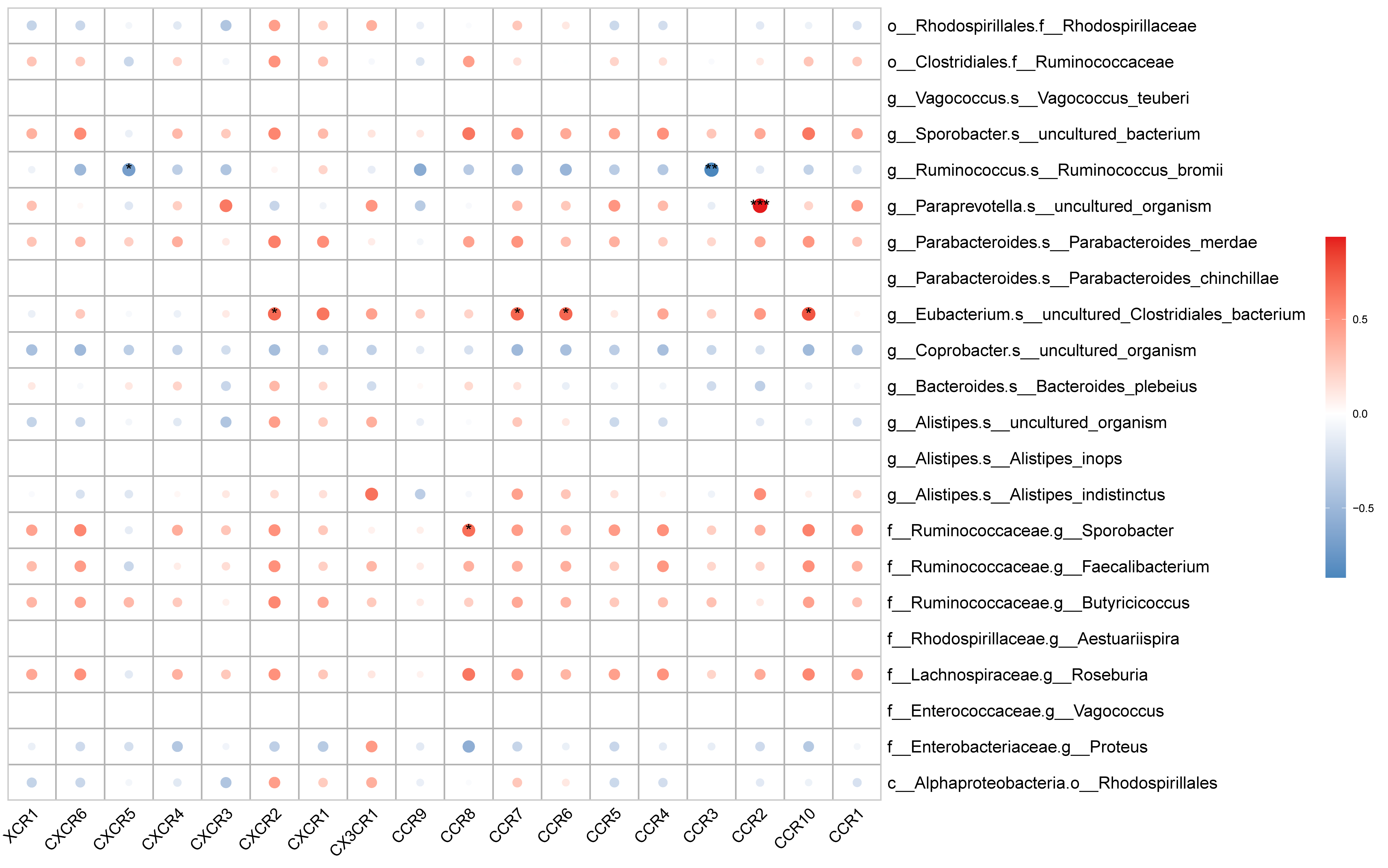

Supplement: Supplementary file 6 — Additional file 6: Figure S6. Heatmap of the correlation between the dominant flora and chemokine receptors in stage III-IV group. The horizontal coordinates indicate genes. The vertical coordinates indicate colonies. Red in the graph represents positive correlation. Blue represents negative correlation. Color depth represents Pearson correlation coefficient size. Color from light to dark indicates Pearson correlation coefficient value from small to large. The "*" in the graph indicates the size of P-value: no * for P-value ≥ 0.05, * for 0.01 ≤ P < 0.05, ** for 0.001 ≤ P < 0.01, *** for P < 0.001. [file 12967_2023_4119_MOESM6_ESM.tif]
