# Supplementary material for: Identification of colorectal cancer progression-associated intestinal microbiome and predictive signature construction
Source: J Transl Med. 2023 Jun 8;21:373. doi: 10.1186/s12967-023-04119-1 (PMC10249256; doi:10.1186/s12967-023-04119-1)
Supplement: Supplementary file 8 — Additional file 8: Table S2. ADONIS test for Jaccard Distance of intestinal flora in patients with early and advanced CRC. Df: degrees of freedom, between-group degrees of freedom are the number of groups minus one, within-group degrees of freedom are the total number of samples minus the number of groups; Group row: between-group statistics; Residuals row: within-group statistics; Total row: between-group plus within-group statistics; Sums Of Sqs: sums of squares of deviations; Mean Sqs: mean square, ratio of sums of squares of deviations to degrees of freedom, i.e., Sums Of Sqs/Df; F.Model: F-test value, i.e., ratio of between-group mean square to within-group mean square; R2: Ratio of between-group and within-group's sums of squares of deviations to the sums of squares of the total deviations, indicating the degree of explanation of the difference between samples, and a larger R2 indicates a higher degree of explanation of the difference between samples; Pr(> F): the statistically significant P value obtained from the substitution test, and Pr < 0.05 is considered statistically significants. [file 12967_2023_4119_MOESM8_ESM.docx]

**Supplementary Table 2. ADONIS test for Jaccard Distance of intestinal flora in patients with early and advanced CRC**

|  | Df | Sums Of Sqs | Mean Sqs | F.Model | R2 | Pr(>F) |
| --- | --- | --- | --- | --- | --- | --- |
| Group | 1 | 0.41560804 | 0.41560804 | 0.965648565 | 0.005056661 | 0.4666 |
| Residuals | 190 | 81.77460257 | 0.430392645 |  | 0.994943339 |  |
| Total | 191 | 82.19021061 |  |  | 1 |  |
