# Supplementary material for: Identification of colorectal cancer progression-associated intestinal microbiome and predictive signature construction
Source: J Transl Med. 2023 Jun 8;21:373. doi: 10.1186/s12967-023-04119-1 (PMC10249256; doi:10.1186/s12967-023-04119-1)
Supplement: Supplementary file 9 — Additional file 9: Table S3. Results of LEfSe analysis. Taxonomy: information of differential species; LDA (log10): effect value of differential species; Group: group with significant abundance of differential species; the table shows species with LDA score (log10) P value less than 0.05 and greater than the present value (default is 2). [file 12967_2023_4119_MOESM9_ESM.docx]

**Additional file 9: Table S3. Results of LEfSe analysis**

| Taxonomy | Group | LDA  (log10) | P value |
| --- | --- | --- | --- |
| g__Vagococcus.s__Vagococcus_teuberi | advanced stage | 3.019479 | 0.003307 |
| f__Enterococcaceae.g__Vagococcus | advanced stage | 3.025048 | 0.003421 |
| g__Eubacterium.s__uncultured_Clostridiales_bacterium | advanced stage | 4.183924 | 0.003795 |
| f__Enterobacteriaceae.g__Proteus | advanced stage | 3.378007 | 0.00417 |
| g__Sporobacter.s__uncultured_bacterium | advanced stage | 3.591634 | 0.006588 |
| f__Ruminococcaceae.g__Sporobacter | advanced stage | 3.715573 | 0.006692 |
| g__Lachnospiracea_incertae_sedis.s__uncultured_bacterium_adhufec37_25 | early stage | 3.149768 | 0.008399 |
| o__Rhodospirillales.f__Rhodospirillaceae | advanced stage | 3.003799 | 0.010904 |
| g__Oscillibacter.s__unidentified_bacterium | early stage | 2.26759 | 0.011696 |
| g__Corynebacterium.s__Corynebacterium_aurimucosum | early stage | 2.715229 | 0.011696 |
| f__Caulobacteraceae.g__Caulobacter | early stage | 3.675239 | 0.011696 |
| g__Parabacteroides.s__Parabacteroides_merdae | advanced stage | 4.161664 | 0.013666 |
| f__Ruminococcaceae.g__Butyricicoccus | advanced stage | 4.399259 | 0.014704 |
| f__Ruminococcaceae.g__Faecalibacterium | advanced stage | 4.851777 | 0.015023 |
| o__Actinomycetales.f__Corynebacteriaceae | early stage | 3.085534 | 0.015253 |
| f__Corynebacteriaceae.g__Corynebacterium | early stage | 3.081032 | 0.015893 |
| g__Coprobacter.s__uncultured_organism | advanced stage | 3.312007 | 0.018697 |
| g__Peptostreptococcus.s__Peptostreptococcus_russellii | early stage | 4.467169 | 0.023937 |
| c__Alphaproteobacteria.o__Rhodospirillales | advanced stage | 3.055356 | 0.024426 |
| g__Parabacteroides.s__Parabacteroides_chinchillae | advanced stage | 2.55214 | 0.024821 |
| o__Clostridiales.f__Ruminococcaceae | advanced stage | 5.244349 | 0.025362 |
| f__Enterobacteriaceae.g__Morganella | early stage | 3.197027 | 0.025751 |
| f__Rhodospirillaceae.g__Aestuariispira | advanced stage | 3.027504 | 0.026398 |
| g__Alistipes.s__uncultured_organism | advanced stage | 3.394767 | 0.026594 |
| g__Alistipes.s__Alistipes_indistinctus | advanced stage | 3.143706 | 0.028364 |
| g__Ruminococcus.s__Ruminococcus_bromii | advanced stage | 4.445491 | 0.032246 |
| g__Bacteroides.s__Bacteroides_plebeius | advanced stage | 4.19705 | 0.032537 |
| g__Alistipes.s__Alistipes_inops | advanced stage | 3.033334 | 0.034363 |
| g__Paraprevotella.s__uncultured_organism | advanced stage | 3.081752 | 0.038645 |
| g__Anaerovorax.s__Eubacterium_sp__oral_clone_FX028 | early stage | 2.974204 | 0.040056 |
| g__Corynebacterium.s__Corynebacterium_sp__canine_oral_taxon_423 | early stage | 3.110688 | 0.040056 |
| g__Bdellovibrio.s__uncultured_Bdellovibrio_sp_ | early stage | 3.800387 | 0.040056 |
| g__Atopobium.s__Atopobium_minutum | early stage | 2.750433 | 0.040056 |
| g__Anaerofustis.s__uncultured_Eubacteriaceae_bacterium | early stage | 3.052922 | 0.040056 |
| f__Carnobacteriaceae.g__Dolosigranulum | early stage | 3.965584 | 0.040056 |
| f__Staphylococcaceae.g__Macrococcus | early stage | 3.777221 | 0.040056 |
| f__Dermatophilaceae.g__Dermatophilus | early stage | 3.82759 | 0.040056 |
| o__Actinomycetales.f__Dermatophilaceae | early stage | 3.665933 | 0.040056 |
| g__Haemophilus.s__Haemophilus_parainfluenzae | early stage | 3.266172 | 0.040056 |
| f__Enterobacteriaceae.g__Citrobacter | early stage | 4.966128 | 0.045172 |
| f__Lachnospiraceae.g__Roseburia | advanced stage | 4.427686 | 0.048434 |
