# Supplementary material for: Identification of colorectal cancer progression-associated intestinal microbiome and predictive signature construction
Source: J Transl Med. 2023 Jun 8;21:373. doi: 10.1186/s12967-023-04119-1 (PMC10249256; doi:10.1186/s12967-023-04119-1)
Supplement: Supplementary file 10 — Additional file 10: Table S4. KEGG functional pathways in the intestinal microbiome of patients with early and advanced CRC. KEGG_Pathway: KEGG pathway; Mean In advanced stage: predicted abundance value of this pathway in each sample in stage III-IV group; Mean In early stage: predicted abundance value of this pathway in each sample in stage I-II group. P value < 0.05 is considered as statistically significant difference. [file 12967_2023_4119_MOESM10_ESM.docx]

**Supplementary Table 4. KEGG functional pathways in the intestinal microbiome of patients with early and advanced CRC**

| KEGG_pathway | Mean In  advanced stage | Mean In  early stage | P value |
| --- | --- | --- | --- |
| ko00590:Arachidonic acid metabolism | 0 | 326814.7554 | 0.011912374 |
| ko00514:Other types of O-glycan biosynthesis | 394380.6238 | 23836.2947 | 0.018208182 |
| ko00472:D-Arginine and D-ornithine metabolism | 114797907.8 | 104126214.6 | 0.115621854 |
| ko04614:Renin-angiotensin system | 364.7239315 | 8664.952795 | 0.140081484 |
| ko04510:Focal adhesion | 0 | 1.656235484 | 0.150750505 |
| ko00601:Glycosphingolipid biosynthesis - lacto and neolacto series | 932584.0947 | 384970.0062 | 0.180626811 |
| ko04142:Lysosome | 17363013.99 | 306634.8013 | 0.190024633 |
| ko00965:Betalain biosynthesis | 13461.10154 | 49823.00323 | 0.195006434 |
| ko05144:Malaria | 11329.30124 | 2460.027576 | 0.207454061 |
| ko04210:Apoptosis | 71196788.31 | 53510400.22 | 0.211863162 |
| ko04974:Protein digestion and absorption | 91348601.76 | 70127884.17 | 0.229657287 |
| ko00523:Polyketide sugar unit biosynthesis | 163380679.8 | 59194756.7 | 0.229877713 |
| ko05146:Amoebiasis | 31788656.99 | 15973736.03 | 0.238389163 |
| ko04020:Calcium signaling pathway | 4581.531338 | 64577.30444 | 0.246495489 |
| ko00340:Histidine metabolism | 1647474918 | 1538874869 | 0.269579346 |
| ko00983:Drug metabolism - other enzymes | 189466175.3 | 486164368.3 | 0.2760624 |
| ko00364:Fluorobenzoate degradation | 28891114.52 | 13839366.72 | 0.287878392 |
| ko05110:Vibrio cholerae infection | 2037493.631 | 60896.74143 | 0.289516395 |
| ko00510:N-Glycan biosynthesis | 83335254.68 | 68947302.64 | 0.291865565 |
| ko02040:Flagellar assembly | 568482590.9 | 540501493.3 | 0.294416119 |
| ko00281:Geraniol degradation | 210936926.5 | 104857997.9 | 0.298162273 |
| ko00941:Flavonoid biosynthesis | 9790726.863 | 13381206.29 | 0.311358154 |
| ko00540:Lipopolysaccharide biosynthesis | 1167192254 | 1190558395 | 0.32345669 |
| ko00670:One carbon pool by folate | 2513982327 | 2362592427 | 0.33307568 |
| ko03020:RNA polymerase | 1409667694 | 1394361577 | 0.334464743 |
| ko02030:Bacterial chemotaxis | 1064362146 | 931005728.1 | 0.335857535 |
| ko04112:Cell cycle - Caulobacter | 2156000817 | 1993275089 | 0.340058275 |
| ko04141:Protein processing in endoplasmic reticulum | 69813256.74 | 65989913.24 | 0.340058275 |
| ko00790:Folate biosynthesis | 1921088398 | 1700007470 | 0.341465973 |
| ko00290:Valine, leucine and isoleucine biosynthesis | 2905420962 | 2888999158 | 0.342877395 |
| ko00770:Pantothenate and CoA biosynthesis | 2446193298 | 2368830562 | 0.342877395 |
| ko00908:Zeatin biosynthesis | 1012416503 | 937965748.7 | 0.345711408 |
| ko03060:Protein export | 2040665177 | 1881002750 | 0.347133996 |
| ko05142:Chagas disease (American trypanosomiasis) | 36524.50692 | 234559.9643 | 0.347274791 |
| ko00900:Terpenoid backbone biosynthesis | 1844310423 | 1691366281 | 0.351424073 |
| ko00400:Phenylalanine, tyrosine and tryptophan biosynthesis | 1579506206 | 1603155974 | 0.352861532 |
| ko00680:Methane metabolism | 753858553.8 | 672630558.9 | 0.352861532 |
| ko00260:Glycine, serine and threonine metabolism | 1494276141 | 1423973301 | 0.354302704 |
| ko00860:Porphyrin and chlorophyll metabolism | 1001462727 | 867354482.6 | 0.357196185 |
| ko01051:Biosynthesis of ansamycins | 5820984884 | 5316408160 | 0.357196185 |
| ko03070:Bacterial secretion system | 1079511892 | 1008898491 | 0.35864849 |
| ko04075:Plant hormone signal transduction | 247755.7797 | 390757.2399 | 0.361293387 |
| ko00970:Aminoacyl-tRNA biosynthesis | 2301272217 | 2154048418 | 0.36156422 |
| ko00720:Carbon fixation pathways in prokaryotes | 1680931010 | 1555066130 | 0.363027642 |
| ko00740:Riboflavin metabolism | 1311256011 | 1142843200 | 0.363027642 |
| ko00780:Biotin metabolism | 2375613513 | 2120128922 | 0.363027642 |
| ko00760:Nicotinate and nicotinamide metabolism | 1636237345 | 1445492343 | 0.364494766 |
| ko03018:RNA degradation | 888061826 | 842732404.8 | 0.364494766 |
| ko00300:Lysine biosynthesis | 2153483928 | 2051946906 | 0.36744011 |
| ko00980:Metabolism of xenobiotics by cytochrome P450 | 21386158.93 | 3721259.356 | 0.36833763 |
| ko00061:Fatty acid biosynthesis | 2196618621 | 2097577204 | 0.370400238 |
| ko00250:Alanine, aspartate and glutamate metabolism | 2311795006 | 2256335670 | 0.37188584 |
| ko00750:Vitamin B6 metabolism | 1644719097 | 1476269436 | 0.37188584 |
| ko03010:Ribosome | 2230378856 | 2083224112 | 0.37188584 |
| ko03030:DNA replication | 1730515466 | 1635132723 | 0.37188584 |
| ko03440:Homologous recombination | 2112244035 | 1989929837 | 0.37188584 |
| ko00471:D-Glutamine and D-glutamate metabolism | 2966540151 | 2661931853 | 0.37337513 |
| ko03410:Base excision repair | 1279298079 | 1224355383 | 0.37337513 |
| ko05120:Epithelial cell signaling in Helicobacter pylori infection | 234023758.8 | 215908218.5 | 0.37337513 |
| ko00550:Peptidoglycan biosynthesis | 2614129714 | 2428256060 | 0.374868108 |
| ko03008:Ribosome biogenesis in eukaryotes | 78728778.06 | 70811957.86 | 0.374868108 |
| ko00660:C5-Branched dibasic acid metabolism | 2314406976 | 2351905586 | 0.379369136 |
| ko00710:Carbon fixation in photosynthetic organisms | 2212190289 | 2092385588 | 0.382388209 |
| ko04626:Plant-pathogen interaction | 255026918.2 | 240229530.4 | 0.382388209 |
| ko00240:Pyrimidine metabolism | 1663557980 | 1548799951 | 0.383903253 |
| ko03013:RNA transport | 79349201.97 | 66236826.17 | 0.383903253 |
| ko04910:Insulin signaling pathway | 122792885.4 | 133976094.8 | 0.383903253 |
| ko00190:Oxidative phosphorylation | 651437972 | 649006246.7 | 0.385421966 |
| ko00730:Thiamine metabolism | 2274229803 | 2136720754 | 0.386944344 |
| ko03430:Mismatch repair | 2387501092 | 2275512047 | 0.386944344 |
| ko03420:Nucleotide excision repair | 1136702907 | 1065955140 | 0.388470385 |
| ko00020:Citrate cycle (TCA cycle) | 1542891211 | 1449030836 | 0.393070454 |
| ko00230:Purine metabolism | 1248788607 | 1164297874 | 0.396155423 |
| ko00270:Cysteine and methionine metabolism | 1663516878 | 1646504488 | 0.397703374 |
| ko00330:Arginine and proline metabolism | 976248980 | 950945263.1 | 0.397703374 |
| ko04621:NOD-like receptor signaling pathway | 117122991.3 | 130079111.5 | 0.400810195 |
| ko00630:Glyoxylate and dicarboxylate metabolism | 937436520.9 | 896782533.5 | 0.402369058 |
| ko04146:Peroxisome | 318899448.5 | 292663571.4 | 0.402369058 |
| ko00280:Valine, leucine and isoleucine degradation | 609403330.6 | 534727179.3 | 0.40393155 |
| ko00440:Phosphonate and phosphinate metabolism | 195351464.2 | 180319157.1 | 0.40393155 |
| ko00620:Pyruvate metabolism | 1589444020 | 1546861529 | 0.407067409 |
| ko01040:Biosynthesis of unsaturated fatty acids | 497052765 | 460393494.8 | 0.408640769 |
| ko00310:Lysine degradation | 279198981.4 | 232504843.1 | 0.410217744 |
| ko00521:Streptomycin biosynthesis | 2521498896 | 2498990735 | 0.410217744 |
| ko04122:Sulfur relay system | 1387896891 | 1283536001 | 0.410217744 |
| ko00640:Propanoate metabolism | 941589679.4 | 876178072.6 | 0.416561713 |
| ko00920:Sulfur metabolism | 1016928597 | 1085338562 | 0.418156703 |
| ko04113:Meiosis - yeast | 351514.1004 | 416994.3698 | 0.418373078 |
| ko00531:Glycosaminoglycan degradation | 1052435046 | 1356959664 | 0.419755284 |
| ko00430:Taurine and hypotaurine metabolism | 966357393.8 | 883387083.4 | 0.421357451 |
| ko01057:Biosynthesis of type II polyketide products | 46916.47115 | 55182.32258 | 0.428085533 |
| ko00473:D-Alanine metabolism | 2353703788 | 2269316648 | 0.429421927 |
| ko00960:Tropane, piperidine and pyridine alkaloid biosynthesis | 504017663.3 | 529618566.5 | 0.429421927 |
| ko00030:Pentose phosphate pathway | 2289882697 | 2126779970 | 0.432672665 |
| ko04970:Salivary secretion | 311550.2182 | 12742.01183 | 0.433979009 |
| ko00564:Glycerophospholipid metabolism | 805464762.8 | 755561577.9 | 0.434303361 |
| ko00071:Fatty acid metabolism | 518365573 | 421711067.5 | 0.435937602 |
| ko00072:Synthesis and degradation of ketone bodies | 482953828.1 | 329780126.7 | 0.439216699 |
| ko05111:Vibrio cholerae pathogenic cycle | 226973995.4 | 188887638 | 0.439216699 |
| ko01055:Biosynthesis of vancomycin group antibiotics | 3382329994 | 3413849002 | 0.440861545 |
| ko00010:Glycolysis / Gluconeogenesis | 1561713769 | 1487411451 | 0.442509916 |
| ko00520:Amino sugar and nucleotide sugar metabolism | 1551697265 | 1567009620 | 0.442509916 |
| ko00450:Selenocompound metabolism | 1471724087 | 1479810949 | 0.445817212 |
| ko03450:Non-homologous end-joining | 23304552.16 | 19977778.53 | 0.445817212 |
| ko00650:Butanoate metabolism | 980857060.3 | 862068109.1 | 0.447476126 |
| ko00600:Sphingolipid metabolism | 694356792.8 | 817710459 | 0.449138544 |
| ko00633:Nitrotoluene degradation | 407930100.7 | 405308750.7 | 0.449138544 |
| ko00561:Glycerolipid metabolism | 647710786 | 614172998 | 0.45080446 |
| ko00052:Galactose metabolism | 1594785979 | 1613487910 | 0.455823143 |
| ko00362:Benzoate degradation | 243315995.9 | 189583576.1 | 0.455823143 |
| ko00910:Nitrogen metabolism | 861944275 | 830608705.7 | 0.465954196 |
| ko00311:Penicillin and cephalosporin biosynthesis | 45741249.06 | 38316324.95 | 0.471066245 |
| ko03040:Spliceosome | 34657.66256 | 61363.31632 | 0.474548944 |
| ko00785:Lipoic acid metabolism | 1729253358 | 1673015099 | 0.476209087 |
| ko00051:Fructose and mannose metabolism | 1456912198 | 1447337160 | 0.483113808 |
| ko00140:Steroid hormone biosynthesis | 82374174.96 | 99585537.92 | 0.484848443 |
| ko00312:beta-Lactam resistance | 328773257.4 | 291486830.1 | 0.484848443 |
| ko00562:Inositol phosphate metabolism | 291617409.3 | 262852374.2 | 0.490072535 |
| ko00360:Phenylalanine metabolism | 398903405.3 | 383724887.4 | 0.491820603 |
| ko02010:ABC transporters | 928972251.6 | 822608633.1 | 0.491820603 |
| ko00500:Starch and sucrose metabolism | 1518920742 | 1511537144 | 0.493572013 |
| ko00350:Tyrosine metabolism | 349911861.8 | 343164248.7 | 0.495326756 |
| ko00460:Cyanoamino acid metabolism | 249244463.2 | 275059532.6 | 0.496282226 |
| ko00331:Clavulanic acid biosynthesis | 14045.69872 | 0 | 0.49684801 |
| ko00563:Glycosylphosphatidylinositol(GPI)-anchor biosynthesis | 4.262721538 | 0 | 0.49684801 |
| ko00909:Sesquiterpenoid biosynthesis | 21389.67192 | 0 | 0.49684801 |
| ko01056:Biosynthesis of type II polyketide backbone | 61221.49872 | 0 | 0.49684801 |
| ko05010:Alzheimer's disease | 4707.807747 | 0 | 0.49684801 |
| ko05014:Amyotrophic lateral sclerosis (ALS) | 1897018.89 | 0 | 0.49684801 |
| ko00627:Aminobenzoate degradation | 223152179.5 | 170707442.7 | 0.497084825 |
| ko00380:Tryptophan metabolism | 262860275.3 | 221489789.4 | 0.498842574 |
| ko02020:Two-component system | 470735147.4 | 414947910.4 | 0.498846213 |
| ko00130:Ubiquinone and other terpenoid-quinone biosynthesis | 629298446.2 | 602804967.1 | 0.509483827 |
| ko00511:Other glycan degradation | 2499512625 | 2926389578 | 0.516640995 |
| ko00943:Isoflavonoid biosynthesis | 13728.48764 | 35330.31221 | 0.518422101 |
| ko00120:Primary bile acid biosynthesis | 165540098.4 | 167589623.2 | 0.518438394 |
| ko05012:Parkinson's disease | 5362454.63 | 1489409.317 | 0.520290574 |
| ko00121:Secondary bile acid biosynthesis | 1483299103 | 1506652136 | 0.525660173 |
| ko00625:Chloroalkane and chloroalkene degradation | 172731390.8 | 302934499.6 | 0.533267426 |
| ko04080:Neuroactive ligand-receptor interaction | 4.088675385 | 0.543459677 | 0.555892619 |
| ko05150:Staphylococcus aureus infection | 41771099.56 | 34901022.06 | 0.560651985 |
| ko00626:Naphthalene degradation | 129266524.3 | 106967855.4 | 0.567719053 |
| ko00040:Pentose and glucuronate interconversions | 913236925.6 | 941484925.7 | 0.57004436 |
| ko04144:Endocytosis | 474500.0612 | 642348.5499 | 0.578547599 |
| ko00361:Chlorocyclohexane and chlorobenzene degradation | 103584793.8 | 82224655.18 | 0.585226343 |
| ko00480:Glutathione metabolism | 669213857 | 581814493 | 0.585226343 |
| ko00621:Dioxin degradation | 288737453.2 | 236917391.7 | 0.590967625 |
| ko05130:Pathogenic Escherichia coli infection | 35451.21498 | 17875.40025 | 0.595680914 |
| ko04962:Vasopressin-regulated water reabsorption | 11916.35991 | 22181.17477 | 0.603305361 |
| ko00903:Limonene and pinene degradation | 45056607.33 | 69219140.46 | 0.617740085 |
| ko05322:Systemic lupus erythematosus | 82348.62687 | 50648.09818 | 0.624481165 |
| ko00624:Polycyclic aromatic hydrocarbon degradation | 3833186.545 | 604006.1678 | 0.648175624 |
| ko00643:Styrene degradation | 65232003.47 | 44368841.96 | 0.649483076 |
| ko05143:African trypanosomiasis | 8980679.581 | 4991005.648 | 0.652745262 |
| ko00053:Ascorbate and aldarate metabolism | 382132827.1 | 339476728 | 0.653751754 |
| ko00930:Caprolactam degradation | 74607118.23 | 29051619.68 | 0.661787524 |
| ko00623:Toluene degradation | 185567365.9 | 45773446.91 | 0.685189864 |
| ko00830:Retinol metabolism | 46219727.14 | 54278871.08 | 0.688257588 |
| ko00410:beta-Alanine metabolism | 646678344.1 | 374829383.6 | 0.700199782 |
| ko00906:Carotenoid biosynthesis | 16204491.17 | 21483665.39 | 0.70047805 |
| ko00513:Various types of N-glycan biosynthesis | 36505.07523 | 409.3703226 | 0.753263622 |
| ko02060:Phosphotransferase system (PTS) | 544148044.8 | 467468821.4 | 0.754689568 |
| ko01053:Biosynthesis of siderophore group nonribosomal peptides | 122067392.4 | 102196225.7 | 0.769507049 |
| ko00196:Photosynthesis - antenna proteins | 93883.30131 | 30756.94931 | 0.798257601 |
| ko00195:Photosynthesis | 147230742.9 | 49312240.48 | 0.800284906 |
| ko04310:Wnt signaling pathway | 799.3638146 | 47.76135484 | 0.840691956 |
| ko00100:Steroid biosynthesis | 18481110.77 | 37996226.16 | 0.849109615 |
| ko00524:Butirosin and neomycin biosynthesis | 172640683.3 | 118581697.2 | 0.859438541 |
| ko03015:mRNA surveillance pathway | 128954.1097 | 47513.07288 | 0.878897762 |
| ko00642:Ethylbenzene degradation | 50377247.98 | 59781606.22 | 0.888111308 |
| ko00363:Bisphenol degradation | 33810381.64 | 115777175.5 | 0.906434487 |
| ko04110:Cell cycle | 12632.18373 | 1631.00186 | 0.914007051 |
| ko03050:Proteasome | 3061825.52 | 1366976.985 | 0.921456612 |
| ko05410:Hypertrophic cardiomyopathy (HCM) | 1152154.511 | 879190.2516 | 0.922380205 |
| ko05145:Toxoplasmosis | 53219.57766 | 139708.057 | 0.931033765 |
| ko05100:Bacterial invasion of epithelial cells | 12605929.24 | 8572720.282 | 0.952382683 |
| ko00791:Atrazine degradation | 122431094 | 80474696.93 | 0.973411073 |
| ko04512:ECM-receptor interaction | 3137.908516 | 948.789171 | 0.974214466 |
| ko00622:Xylene degradation | 44856699.19 | 58112836.1 | 0.974362034 |
| ko00591:Linoleic acid metabolism | 160346243.5 | 220273299.1 | 0.992170169 |
