# Supplementary material for: Identification of colorectal cancer progression-associated intestinal microbiome and predictive signature construction
Source: J Transl Med. 2023 Jun 8;21:373. doi: 10.1186/s12967-023-04119-1 (PMC10249256; doi:10.1186/s12967-023-04119-1)
Supplement: Supplementary file 11 — Additional file 11: Table S5. List of differential GO items of CRC patients stratified by CRC progression. GO items: Enriched GO entries. FC in logFC, i.e., fold change, indicates the ratio of expression in stage III-IV CRC group to that in stage I-II CRC group and is taken as logarithm with a base of 2. P.value < 0.05 is taken as statistically significant difference. [file 12967_2023_4119_MOESM11_ESM.docx]

**Additional file 11: Table S5. List of differential GO items of CRC patients stratified by CRC progression**

| GO items | logFC | P.Value |
| --- | --- | --- |
| GOBP_POSITIVE_REGULATION_OF_INTEGRIN_ACTIVATION | -0.05338 | 0.002939 |
| GOBP_POSITIVE_REGULATION_OF_HETEROTYPIC_CELL_CELL_ADHESION | -0.04011 | 0.006853 |
| GOBP_POLYAMINE_BIOSYNTHETIC_PROCESS | -0.0322 | 0.007086 |
| GOBP_PURINE_DEOXYRIBONUCLEOTIDE_CATABOLIC_PROCESS | -0.04134 | 0.007334 |
| GOBP_NEGATIVE_REGULATION_OF_TOLL_LIKE_RECEPTOR_4_SIGNALING_PATHWAY | -0.05289 | 0.008821 |
| GOBP_REGULATION_OF_HYDROGEN_PEROXIDE_METABOLIC_PROCESS | 0.029769 | 0.011429 |
| GOBP_RESPONSE_TO_GRAVITY | -0.05144 | 0.011909 |
| GOBP_PROTEIN_FOLDING_IN_ENDOPLASMIC_RETICULUM | 0.030965 | 0.013281 |
| GOBP_CELLULAR_TRIVALENT_INORGANIC_ANION_HOMEOSTASIS | 0.049753 | 0.013497 |
| GOBP_REGULATION_OF_DENDRITIC_SPINE_MAINTENANCE | -0.03799 | 0.014813 |
| GOBP_CORNEA_DEVELOPMENT_IN_CAMERA_TYPE_EYE | 0.030669 | 0.016189 |
| GOBP_REGULATION_OF_DOPAMINERGIC_NEURON_DIFFERENTIATION | -0.0789 | 0.017745 |
| GOBP_NEGATIVE_REGULATION_OF_PROTEIN_EXIT_FROM_ENDOPLASMIC_RETICULUM | 0.029819 | 0.01797 |
| GOBP_REGULATION_OF_PROTEIN_EXIT_FROM_ENDOPLASMIC_RETICULUM | 0.026782 | 0.018107 |
| GOBP_GLUCOSE_6_PHOSPHATE_METABOLIC_PROCESS | -0.02325 | 0.018442 |
| GOBP_GROOMING_BEHAVIOR | -0.03246 | 0.018959 |
| GOBP_MALE_GENITALIA_DEVELOPMENT | 0.020985 | 0.02045 |
| GOBP_CELL_VOLUME_HOMEOSTASIS | -0.0214 | 0.020553 |
| GOBP_INTERCELLULAR_TRANSPORT | -0.0389 | 0.021236 |
| GOCC_PRC1_COMPLEX | 0.031533 | 0.022121 |
| GOBP_REGULATION_OF_CYSTEINE_TYPE_ENDOPEPTIDASE_ACTIVITY_INVOLVED_IN_APOPTOTIC_SIGNALING_PATHWAY | -0.03062 | 0.022871 |
| GOBP_NEGATIVE_REGULATION_OF_VIRAL_LIFE_CYCLE | -0.02824 | 0.023947 |
| GOCC_SYMMETRIC_SYNAPSE | -0.04246 | 0.024283 |
| GOBP_REGULATION_OF_INTEGRIN_ACTIVATION | -0.03443 | 0.025515 |
| GOMF_DELTA_CATENIN_BINDING | -0.03138 | 0.025912 |
| GOBP_POLYAMINE_METABOLIC_PROCESS | -0.02917 | 0.025996 |
| GOBP_POSITIVE_REGULATION_OF_OXIDATIVE_STRESS_INDUCED_CELL_DEATH | 0.024836 | 0.02675 |
| GOBP_MITOTIC_DNA_REPLICATION | -0.04854 | 0.02749 |
| GOBP_NEGATIVE_REGULATION_OF_MYOBLAST_DIFFERENTIATION | -0.02522 | 0.027827 |
| GOMF_BHLH_TRANSCRIPTION_FACTOR_BINDING | -0.02169 | 0.028719 |
| GOBP_REGULATION_OF_MICROTUBULE_BINDING | 0.02317 | 0.031505 |
| GOCC_GOLGI_TRANSPORT_COMPLEX | 0.026954 | 0.031878 |
| GOBP_POSITIVE_REGULATION_OF_CYSTEINE_TYPE_ENDOPEPTIDASE_ACTIVITY_INVOLVED_IN_APOPTOTIC_SIGNALING_PATHWAY | -0.03047 | 0.032373 |
| GOBP_RESPONSE_TO_AMINE | -0.02057 | 0.033087 |
| GOBP_TRAIL_ACTIVATED_APOPTOTIC_SIGNALING_PATHWAY | -0.03316 | 0.03466 |
| GOBP_RESPONSE_TO_THYROID_HORMONE | -0.01855 | 0.035138 |
| GOMF_PEPTIDYL_PROLINE_DIOXYGENASE_ACTIVITY | -0.029 | 0.035547 |
| GOMF_PROTEIN_DISULFIDE_REDUCTASE_NAD_P_ACTIVITY | -0.02447 | 0.036607 |
| GOBP_NATURAL_KILLER_CELL_MEDIATED_IMMUNE_RESPONSE_TO_TUMOR_CELL | -0.04558 | 0.036614 |
| GOBP_TRIVALENT_INORGANIC_ANION_HOMEOSTASIS | 0.04085 | 0.036664 |
| GOMF_PROTON_CHANNEL_ACTIVITY | -0.04161 | 0.037574 |
| GOMF_VITAMIN_BINDING | 0.02043 | 0.037859 |
| GOMF_PROTEIN_TYROSINE_THREONINE_PHOSPHATASE_ACTIVITY | -0.06058 | 0.03916 |
| GOBP_LIPID_DROPLET_FORMATION | 0.026805 | 0.039174 |
| GOBP_RELEASE_OF_CYTOCHROME_C_FROM_MITOCHONDRIA | -0.01836 | 0.039196 |
| GOBP_RESPONSE_TO_INTERLEUKIN_6 | -0.02401 | 0.039452 |
| GOBP_PROTON_TRANSPORTING_TWO_SECTOR_ATPASE_COMPLEX_ASSEMBLY | 0.031763 | 0.039607 |
| GOMF_MAP_KINASE_TYROSINE_SERINE_THREONINE_PHOSPHATASE_ACTIVITY | -0.04673 | 0.041255 |
| GOBP_DEOXYRIBONUCLEOTIDE_CATABOLIC_PROCESS | -0.02333 | 0.041257 |
| GOMF_MOLECULAR_FUNCTION_ACTIVATOR_ACTIVITY | -0.02133 | 0.041596 |
| GOBP_REFLEX | -0.03138 | 0.043648 |
| GOBP_NEGATIVE_REGULATION_OF_FIBROBLAST_PROLIFERATION | -0.01546 | 0.044018 |
| GOBP_PSEUDOPODIUM_ORGANIZATION | -0.029 | 0.044141 |
| GOBP_REGULATION_OF_VIRAL_ENTRY_INTO_HOST_CELL | -0.02086 | 0.044284 |
| GOBP_POSITIVE_REGULATION_OF_CELL_ADHESION_MEDIATED_BY_INTEGRIN | -0.05002 | 0.044328 |
| GOBP_LIPID_DROPLET_ORGANIZATION | 0.019703 | 0.04498 |
| GOBP_GENITALIA_DEVELOPMENT | 0.022626 | 0.045245 |
| GOBP_POSITIVE_REGULATION_OF_CHROMATIN_BINDING | -0.03096 | 0.046317 |
| GOBP_MANNOSYLATION | 0.021316 | 0.046571 |
| GOMF_PHOSPHATIDYLINOSITOL_PHOSPHATE_5_PHOSPHATASE_ACTIVITY | 0.017414 | 0.046982 |
| GOBP_POLYOL_TRANSMEMBRANE_TRANSPORT | -0.04028 | 0.047282 |
| GOBP_ACTIVATION_OF_GTPASE_ACTIVITY | -0.0181 | 0.0481 |
| GOMF_DEATH_RECEPTOR_ACTIVITY | -0.0326 | 0.048539 |
| GOBP_MESENCHYMAL_STEM_CELL_DIFFERENTIATION | -0.02691 | 0.048663 |
| GOMF_GLUTAMATE_BINDING | -0.02988 | 0.049478 |
