# Supplementary material for: Identification of colorectal cancer progression-associated intestinal microbiome and predictive signature construction
Source: J Transl Med. 2023 Jun 8;21:373. doi: 10.1186/s12967-023-04119-1 (PMC10249256; doi:10.1186/s12967-023-04119-1)
Supplement: Supplementary file 12 — Additional file 12: Table S6. List of differential KEGG pathways of CRC patients stratified by CRC progression. KEGG pathway: enriched KEGG pathway. FC in logFC, fold change, i.e., indicates the ratio of expression in stage III-IV CRC group to that in stage I-II CRC group and is taken as logarithm with a base of 2. P.value < 0.05 is taken as statistically significant difference. [file 12967_2023_4119_MOESM12_ESM.docx]

**Additional file 12: Table S6. List of differential KEGG pathways of CRC patients stratified by CRC progression**

| KEGG pathway | logFC | P.Value |
| --- | --- | --- |
| KEGG_OTHER_GLYCAN_DEGRADATION | 0.031371 | 0.007245 |
| KEGG_STEROID_BIOSYNTHESIS | 0.088771 | 0.022653 |
| KEGG_GALACTOSE_METABOLISM | -0.02053 | 0.028949 |
